# Characterization of Essential Oils and Ethanolic Extracts from Nine Pepper Species: Antioxidant and Antimicrobial Activity and Spectroscopic Analysis

**DOI:** 10.3390/molecules30204140

**Published:** 2025-10-20

**Authors:** Aleksandra Sander, Maja Bival Štefan, Tea Sander, Dajana Kučić Grgić, Jelena Parlov Vuković, Iva Blažević, Jasna Jablan

**Affiliations:** 1 Faculty of Chemical Engineering and Technology, University of Zagreb, Trg Marka Marulića 19, 10000 Zagreb, Croatiadkucic@fkit.unizg.hr (D.K.G.); iblazevic@fkit.unizg.hr (I.B.); 2 Faculty of Pharmacy and Biochemistry, University of Zagreb, Trg Marka Marulića 20, 10000 Zagreb, Croatia; maja.bival@pharma.unizg.hr (M.B.Š.); jasna.jablan@pharma.unizg.hr (J.J.); 3 NMR Centre, Ruđer Bošković Institute, Bijenička cesta 54, 10000 Zagreb, Croatia

**Keywords:** pepper species, GC-MS, FTIR, ^1^H NMR, TXRF, antioxidant, antimicrobial, multielement analysis

## Abstract

This study examined the characteristics of essential oils and ethanolic extracts from nine pepper species’ fruits to determine their chemical compositions and assess their biological activity. Ethanolic extracts and essential oils were analyzed using HPLC, GC-MS, FTIR, and ^1^H NMR spectroscopy. The total phenolic content, total flavonoid content, antioxidant activity (DPPH assay), and antibacterial efficacy against five bacterial strains were assessed. Additionally, multielement analysis was performed using the TXRF method. The results demonstrated that the yields and chemical compositions differed markedly according to the pepper origin and extraction method. Ethanolic extracts consistently demonstrated greater total phenolic content and total flavonoid content and enhanced antioxidant and antibacterial properties relative to their respective essential oils. The increased bioactivity is due to the presence of non-volatile, polar compounds, which are not effectively transferred via hydrodistillation. Piperine was solely detected in extracts from black, green, white, Bengali, and Voatsiperifery peppers. This study emphasizes the necessity of optimizing extraction techniques to enhance the bioactivity of pepper extracts, highlighting their potential as sources of natural antioxidants and antibacterial agents.

## 1. Introduction

Pepper has been used since ancient times all over the world and plays an important role as a spice and as a traditional medicine [1]. Although the term “pepper” most often refers to one or two species, mainly *Piper nigrum* L. and *Piper longum* L., it encompasses many species that do not necessarily belong to the genus Piper. Within this study, we have evaluated nine pepper species: black, green, and white pepper (all three originating from *P. nigrum* but processed differently); Bengali pepper (*P. longum*); Voatsiperifery pepper (*Piper borbonense* (Miq.) C.DC.); Javanese pepper (*Piper cubeba* L.f.)—all belonging to the Piperaceae family—and Melegueta pepper (*Aframomum melegueta* K. Schum.), Sichuan pepper (*Zanthoxylum simulans* Hance), and pink pepper (*Schinus terebinthifolia* Raddi), belonging to the Zingiberaceae, Rutaceae, and Anacardiaceae families, respectively.

Peppers are rich in bioactive components, which contribute to their antioxidant, antimicrobial, anticancer, and anti-inflammatory properties. The chemical composition of peppers is diverse, containing alkaloids, essential oils, phenolic compounds, and minerals [2]. Piperine is an alkaloid found as the main component of pepper, mostly characteristic of the Piperaceae family. It is known for its ability to increase the bioavailability of drugs and, as such, is of interest to the pharmaceutical industry [3]. The extraction of pepper bioactive components with ethanol is an optimal choice due to its neutral nature and ability to extract a wide range of compounds, including hydrophilic and lipophilic components [4]. Some pepper ethanolic extracts have been reported as antioxidants [5,6,7,8,9] and antimicrobials [10]. However, there is a lack of comparative studies of different pepper species under the same extraction conditions, which would allow a comparison of their biological activity.

On the other hand, essential oils are complex natural mixtures composed of volatile organic compounds belonging to different chemical families. Pepper species most commonly contain sesquiterpene alcohols, monoterpenes, chromenes, benzoic acid derivatives, and arylpropanoids [11].

The essential oil industry is developing rapidly, with essential oils appearing in many products on the market, from food to cosmetics to nutritional supplements and herbal medicines. Since essential oils come from nature, many people consider them safe to use. Essential oils have been shown to have anti-inflammatory, antioxidant, antibacterial, and antiviral properties and positive effects on stress, cardiovascular, and neurological problems. Although most essential oils are safe to use, toxic reactions have been reported [12,13]. However, research on essential oils has not yet yielded conclusive results regarding their use, which indicates that additional scientific research is needed to verify their composition and potential effects [12].

Verification of authenticity and compliance with quality standards is mandatory in the quality control of plant extracts and essential oils. Quality control also implies the use of analytical methods that are environmentally sustainable, fast, simple, and low-cost [14]. The chemical composition of essential oils varies with climatic and geographical factors, as well as the extraction methods, making the analysis process challenging and complicated. A common method of analyzing essential oils is gas chromatography coupled with mass spectroscopy (GC-MS), which provides detailed insights into the composition of the essential oil but requires time, skill, and experience. Ethanolic extracts are usually characterized by high-performance liquid chromatography (HPLC), as a fast, automated, and precise analytical technique, but certain disadvantages, such as the cost, high consumption of solvents, and complicated method development, are still associated with this technique [15]. For these reasons, research is focused on the use of simple analytical spectroscopic methods such as Fourier-transform infrared spectroscopy (FTIR) as a fast, green, non-destructive, and cost-effective technique for ethanolic extracts’ and essential oils’ quality assessment [16]. Nuclear magnetic resonance spectroscopy (NMR) is a powerful technique for identifying components in complex mixtures. Due to its speed and strong capabilities, it enables the verification of the identity, purity, and composition, which is the basis of quality control analysis [17]. Additionally, no other analytical technique achieves the same power in structural elucidation [18].

Through this research, a comparative overview of the ethanolic extract and essential oil compositions of different plant species colloquially called pepper is provided. One of the aims was to analyze volatile compounds using GC-MS as the technique of choice, with the additional evaluation of the potential of FTIR and ^1^H NMR spectroscopy as rapid and reliable techniques in the analysis of components with a complex matrix. Secondly, the content of piperine in ethanolic extracts was determined using HPLC, while FTIR and ^1^H NMR spectroscopy were also investigated as potential techniques for detecting the presence of piperine. Both essential oils and ethanolic extracts were evaluated for their polyphenolic content. The next goal was to assess the antioxidant and antimicrobial activity of pepper essential oils and ethanolic extracts, since such activity is one of the prerequisites for their future application in the food and pharmaceutical industries. Finally, the multielement compositions of the tested plant species were analyzed. Considering the comprehensive characterization of diverse peppers and the evaluation of their biological effects, this study provides substantive insights into the potential applications of selected pepper species.

## 2. Results and Discussion

### 2.1. Essential Oil and Ethanolic Extract Yields

To enable the comparison of the experimental data acquired in this study, Table 1 presents both the experimental data and the reported minimum and maximum yields of essential oils and ethanolic extracts. The table exclusively presents published data for essential oils produced by hydrodistillation (HD) and ethanolic extracts obtained using UAE utilizing 70% ethanol. The yields of essential oils and ethanolic extracts for the majority of the peppers examined in this study match the existing literature data (Table 2 and Table 3). The yields of essential oils and ethanolic extracts from various peppers exhibit considerable variability due to their diverse origins. The reported range of ethanolic extracts (Table 3) is directly affected by the extraction method (maceration, Soxhlet, microwave or ultrasound-assisted, supercritical fluid) [5,6,9,19] and the purity of the ethanol [20,21]. Under similar process conditions (70% ethanol and UAE), data comparable to the findings of this investigation are available solely for black pepper.

**Table 1:** Yields of essential oils and ethanolic extracts.

Pepper Species	Essential Oil Yield, %		Refs.	Ethanolic Extract Yield, %		Ref.
	Exp	Lit ^1^		Exp	Lit ^2^	
Black pepper	2.18	0.91–3.68	[22,23]	12.5	9.80	[5]
Green pepper	1.86	0.47–3.76	[24,25]	12.6	-	-
White pepper	2.44	0.44–4.12	[22,23]	6.4	-	-
Melegueta pepper	0.27	0.21–0.30	[26,27,28]	4.8	-	-
Voatsiperifery pepper	6.09	3.04–11.30	[29,30]	18.0	-	-
Javanese pepper	9.73	0.20–11.80	[21,31]	17.4	-	-
Pink pepper	6.63	0.16–6.54	[32,33]	41.0	-	-
Bengali pepper	0.86	0.10–0.80	[34,35]	27.71	-	-
Sichuan pepper	3.93	-	-	26.6	-	-

^1^ Lowest and highest values reported. ^2^ UAE with 70% ethanol.

Table 2 and Table 3 provide an overview of the available literature related to the pepper essential oils and ethanolic extracts investigated in this study. In addition to the extraction method, the yields and chemical compositions (major components) of the essential oils and the yields of ethanolic extracts and piperine are shown. Most of the pepper essential oils investigated in this work are sufficiently represented in the literature, and the values obtained in this work can be compared with previously published data. The smallest number of works was found for Melegueta and Voatsiperifery peppers, while no data on the yield and chemical composition of the essential oil were found for Sichuan pepper. The most commonly used method of extracting essential oils is hydrodistillation (Clevenger), which varies in duration from one process to another. Recently, the application of ultrasound and microwaves (microwave-assisted hydrodistillation (MAHD); ultrasound-assisted hydrodistillation (UAHD); and ultrasonic and microwave-assisted hydrodistillation (UMAHD)) has been investigated with the aim of increasing the efficiency of the extraction of essential oils from black, green, and white pepper. Some authors have used commercial essential oils in their research, and the least common method is steam distillation. The observed differences in the yield and composition of essential oils (Table 2) are mainly a consequence of the extraction method used and the geographical origin of the raw material. Wang et al. [36] investigated the influence of ultrasound and microwaves on the essential oil yield, superoxide radical scavenging activity, and chemical composition of white pepper essential oil. They concluded that the combined utilization of ultrasound and microwaves enhances the extraction of phytochemicals, resulting in higher yields and higher-quality essential oils. Among the applied methods, UAHD was the least efficient. All extracts had better antioxidant activity than BHT. These findings suggest that optimizing the extraction techniques can significantly impact the quality and potency of essential oils derived from various plant sources. Future research may explore additional methods or combinations to further improve the extraction efficiency and the bioactivity of essential oils.

**Table 2:** Overview of the literature data on the topic of the yields and major compounds in pepper species essential oils.

Pepper Species	Extraction Method	Yield, %	Major Compounds	Ref.
Black pepper	Commercial oil	-	(E)-caryophyllene (27.404%); limonene (14.74%); β-phellandren (10.69%); pinene (7.73%)	[37]
	HD—6 h	1.69–3.68	β-caryophyllene, α-pinene, β-pinene, sabinene, 3-carene, and limonene	[22]
	HD—4 h	0.905	α-pinene (6.61%), β-pinene (15.87%), 3-carene (17.57%), limonene (35.6%), β-caryophyllene (9.48%)	[23]
	Commercial oil	-	β-caryophyllene (30.33%), limonene (12.12%), sabinene (7.52%), β-pinene (7.42%)	[38]
	UMAHD	4.00	α-pinene (8.6%), β-pinene (14.0%), 3-δ-carene (33.2%), limonene (19.2%), caryophyllene (13.0%)	[36]
	HD—5 h	0.359–2.079	3-carene (6.2–26.84%), limonene (4.39–25.83%), caryophyllene (25.58–62.23%), (1R)-2,6,6-trimethylbicyclo[3.1.1]hept-2-ene (0–40.85%)	[39]
	HD	1.98–3.57	β-pinene (5.4–7.2%), α-phellandrene (11.4–18.2%), limonene (15.9–20.0%), β-caryophyllene (9.5–15.9%)	[25]
	HD—7 h	1.11	β-caryophyllene (51.12%), β-thujene (20.58%)	[40]
Green pepper	Commercial oil	-	β-pinene (24.42%), δ3-carene (19.72%), limonene (18.73%), α-pinene (10.39%)	[38]
	HD	2.76–3.76	β-pinene (6.2–7.4%), α-phellandrene (11.8–14.7%), limonene (16.4–19.1%), β-caryophyllene (10.0–16.3%)	[25]
	HD—3 h	0.75	3-carene (35.21%), D-limonene (21.54%), β-caryophyllene (10.05%), β-pinene (9.17%), sabinene (7.37%)	[41]
White pepper	HD—6 h	1.68–4.12	β-caryophyllene, α-pinene, β-pinene, sabinene, 3-carene, and limonene	[22]
	HD—4 h	0.44	α-pinene (7.31%), β-pinene (16.18%), 3-carene (18.02%), limonene (26.03%), β-caryophyllene (14.42%)	[23]
	UAHD, MAHD, UMAHD	3.4–4.1	β-pinene (6.9–9.3%), 3-δ-carene (23.1–25.1%), limonene (15.9–23.2%), caryophyllene (25.1–33.4%)	[36]
	HD—5 h	0.538–2.25	3-carene (0–25.09%), limonene (8.77–20.84%), caryophyllene (43.96–58.24%), (1R)-2,6,6-trimethylbicyclo[3.1.1]hept-2-ene (0–17.39%)	[39]
	HD	2.25–2.92	β-pinene (6.4–7.0%), α-phellandrene (10.3–11.5%), limonene (17.0–18.9%), β-caryophyllene (16.2–17.3%)	[25]
	HD—6 h	-	sabinene (12.6%), β-pinene (7.3%), limonene (11.9%), β-bisabolene (7.4%), torreyol (9.3%)	[42]
	SD—6 h	-	α-pinene (5.20–10.65%), sabinene (0.14–21.58%), β-pinene (8.18–14.82%), Δ-3-carene (21.37–27.83%), DL-limonene (15.41–21.68%), caryophyllene (6.99–30.90%)	[43]
Melegueta pepper	HD	0.30	α-caryophyllene (48.78%), β-caryophyllene (32.50%), linalool (5.40%), E-nerolidol (4.33%)	[26]
	HD—3 h	0.21	α-humulene (60.9%), β-caryophyllene (21.7%), humulene oxide II (5.5%)	[27]
	HD—4 h	0.30	humulene (16.30%), gingerol (15.40%), gingerone (24.27%), gingerdione (22.46%)	[28]
Voatsiperifery pepper	HD	3.04	limonene (27.31%), α-phellandrene (14.47%), asaricin (13.47%), β-pinene (6.81%), α-pinene (6.78%)	[29]
	HD	11.6	limonene + eucalyptol (29.54%), α-phellandrene (14.38%), asaricin (13.94%), β-pinene (6.46%), α-pinene (6.00%)	[30]
Javanese pepper	HD—4 h	1.01	β-myrcene (21.19%), 1,8-cineole (6.41%), eugenol (10.66%)	[23]
	HD—4 h	11.8	sabinene (9.1%), β-elemene (9.4%), β-caryophyllene (3.1%), epi-cubebol (4.3%), cubebol (5.6%)	[31]
	HD—3 h	9.6	sabinene (46.3%), 4-terpineol (17.0%), γ-terpinene (4.2%)	[44]
	HD—3 h	2.4	β-cubebene (18.94%), cubebol (13.32%), sabinene (9.60%), α-copaene (7.41%), β-caryophyllene (5.28%)	[45]
	HD—7 h	1.23	terpinen-4-ol (42.41%), α-copaene (20.04%), γ-elemene (17.68%)	[40]
	HD—4 h	2.3	methyleugenol (41.31%), eugenol (33.95%), (E)-caryophyllene (5.65%)	[46]
	HD	2.3	sabinene (19.4%), β-cubebene (18.3%), α-copaene (8.8%), β-phellandrene (5.9%)	[47]
Pink pepper	HD—2 h	-	β-myrcene (41%), 218 β-cubebene (12%), limonene (9%), α-pinene (8%)	[48]
	HD—3 h	2.93	α-pinene (14.22%), sabinene (31.39%), β-myrcene (7.83%), α-phellandrene (11.27%), β-phellandrene (7.57%), germacrene D (8.62%)	[49]
	HD	1.77–4.77	α-pinene (20.7–57%), δ-3-carene (11.07–17%), cis-ocimene (3.3–27.9%), p-cymene (2.6–7.1%), limonene (8–11%)	[50]
	HD—6 h	6.54 ± 1.06	α-phelandrene (35.84%), limonene (17.31%), α-pinene (1.98%), monoterpenes and β-phelandrene (13.04%)	[33]
	HD	0.16	limonene (16.99%), germacrene D (10.85%), δ-cadinene (9.21%), myrcene (20.43%)	[32]
Bengali pepper	HD—4 h	0.285	β-caryophyllene (11.85%), α-humulene (6.25%), 1-heptadecene (11.03%), n-heptadecane (11.93%)	[23]
	HD—4 h	0.49	(Z)-β-farnesene (25.08%), β-caryophyllene (13.57%), α-humulene (13.37%), 8-heptadecene (9.28%), heptadecane (7.07%)	[51]
	HD—6 h	0.10	α-pinene (15.3%), β-pinene (43.1%), limonene (9.6%), nerolidol (8.8%)	[34]
	SD	1.01	caryophyllene (10.78%), 3-heptadecene (9.95%), zingiberene (9.54%), germacrene D (8.96%), pentadecane (8.76%), heptadecane (8.73%)	[52]
	SD	-	(n)-trans-nerolidol (12.7%), β-linalool (8,4%)	[53]
	HD	0.15–0.80	β-caryophyllene (15–25%), hexadecen-1-ol (3.75%), α-caryophyllene (9.58%), β-humulene (6.17%), pentadecane (6.48%)	[35]

Significant variations in yield and composition (e.g., the amount of piperine in the extract) can be observed in published studies regarding the production of ethanolic extracts of pepper (Table 3). The various pepper origins, the extraction process, and the purity (30–99.9%) of the ethanol employed all contribute to these findings. Extracts are derived through maceration (MAC), reflux extraction (RE), Soxhlet extraction (SE), mixing, supercritical fluid extraction, pressurized liquid extraction (PLE), percolation, and methods enhanced by microwaves (MAE), ultrasound (UAE), or their combination (UMAE). Microwaves and ultrasound are frequently employed to enhance the extraction efficiency; nevertheless, both techniques elevate the temperature, potentially leading to the decomposition (thermal degradation or oxidation) of heat-sensitive compounds, including phenols and flavonoids. The effects of the extraction method, ethanol concentration, solvomodul, temperature, and time on black pepper ethanolic extract yields and antioxidative activity have been investigated by Milenković et al. [5]. To determine the optimal process conditions (70% ethanol and solvomodul 1:10, m/V), ethanolic extracts were produced by maceration (*T* = 25 °C, *t* = 120 min). The yield increased with elevated temperatures and prolonged extraction durations. The maximum yield was achieved by SE (240 min), while the minimum yield was achieved by UAE (60 min). The impacts of various solvents (ethanol, methanol, acetone, and dichloromethane), extraction techniques (MAE, UAE, UMAE, SE), and process conditions (particle size, solvent-to-solid ratio, MAE power and duration, UAE temperature and duration) on the yield of piperine in the resulting black pepper extracts have been investigated by Gorgani et al. [54]. The maximum yield among the tested solvents was achieved with ethanol and the finest black pepper particles. Increasing the solvent-to-solid ratio from 5:1 to 20:1 enhanced the yield of piperine in ethanolic extracts. The optimal process conditions for UMAE have been determined based on the results obtained. Relative to SE, MAE, and UAE, UMAE emerged as the most efficient technique for piperine extraction from black pepper. A comparable study was carried out by Zhang et al. [6]. They evaluated the quality of green pepper ethanolic extracts derived from MAE, UAE, MAE, and UMAE methods. According to the extensive evaluation of the oleoresin, piperine, and phenolic content, it was determined that UMAE yielded the highest-grade oleoresin. However, the largest oleoresin yield was achieved using UAE, although the oleoresin produced using MAE exhibited the highest total phenolic content and antioxidative activity (DPPH and ABTS). The effect of the extraction process on the yield of oleoresin or piperine has been studied for pink and Bengal pepper, respectively. Andrade et al. [19] investigated the impacts of the extraction methods (SFE, SE, and UAE) and solvents (hexane, ethyl acetate, ethanol) on the oleoresin yield and antioxidative activity (TPC, DPPH) of pink pepper. The highest yield and antioxidative activity of oleoresin were achieved through SE using ethanol. The TPC was slightly higher when ethyl acetate was used. Dias et al. [9] investigated the influence of the temperature and extraction duration on the global extraction yield, chemical composition, and antioxidative activity of pink pepper ethanolic extracts obtained using PLE, comparing the results with those obtained using SE. The highest-quality extract was produced through PLE at an elevated temperature and extended extraction duration. SE was proven to be more a efficient method than UAE regarding the yield, TPC, TFC, and antioxidative activity [55]. Rathod and Rathod [56] have studied the impacts of extraction methods (MIX, UAE, SE) and solvents (acetone, hexane, ethanol) on the yield of piperine from Bengal pepper. Since UAE was more efficient, the influence of the process conditions was additionally investigated. The optimal process conditions were as follows: ethanol as the extracting solvent, an extraction duration of 18 min, a solid-to-solvent ratio of 1:10, ultrasound power of 125 W, an 80% duty cycle, an ultrasonic frequency of 25 kHz, and a temperature of 50 °C. The influence of the ethanol concentration on the yield of the extract and piperine was investigated for white and Javanese pepper. Le et al. [57] studied the impacts of the process conditions (ethanol concentration, solvent-to-solute ratio, temperature, extraction duration, used volume of extractor, and extraction cycles) on the piperine yield in ethanolic extracts of white pepper obtained using percolation. The optimal process conditions were as follows: ethanol concentration (85% *v*/*v*); solvent/solid ratio (3.4:1 mL/g), and extraction duration (78 min). Dwita et al. [21] found that a 70% ethanol concentration yielded a greater amount of Javanese pepper extract than a 96% ethanol concentration. There are no published findings on ethanol extracts for Sichuan and Voatsiperifery peppers.

The results obtained by UAE in this research are comparable to the yields obtained using MAC, MAE, PLE, SE, and PER. It must be emphasized that UAE is a fast, affordable, and environmentally friendly technique that does not require the use of expensive and demanding equipment or the consumption of gas. From the results, it can be concluded that the yields for most pepper species were high and therefore satisfactory for potential use in the pharmaceutical industry. White and Melegueta pepper are the only two types for which it would be advisable to test other extraction techniques in order to obtain a higher extraction yield.

Variations in the extraction methods, processing conditions, and sources of peppers utilized for essential oil and ethanolic extract production significantly complicate the comparison of the existing literature and the findings of this study. Essential oils and ethanolic extracts with varying chemical compositions will exhibit distinct antioxidant and antibacterial activity.

**Table 3:** Overview of the literature data on the topic of the extract yield (EE) and piperine yield (pip) in pepper ethanolic extracts.

Pepper Species	Extraction Method	Yield (EE), %	Yield (Pip), %	Ref.
Black pepper	RE with 96% ethanol	9.8	-	[8]
	MAC, RE, UAE, SE; results are given for UAE with 70% ethanol	9.8	-	[5]
	Mixing, ethanol purity not specified	10–12.48	21–37.50	[58]
	MAE, UAE, UMAE, SE with >95% ethanol—results are given for UAE	-	3.70	[54]
	Shaking with 80% ethanol	-	3.29–7.39	[59]
	SE with 96% ethanol	7.12		[60]
	MAE with 96% ethanol	16.28	5.41	[61]
Green pepper	Shaking with 80% ethanol	-	5.09–8.61	[59]
	MAC, UAE, MAE, UMAE with anhydrous ethanol—results are given for UAE	Up to 11.4	up to 19.25	[6]
White pepper	MAC in 96% ethanol	-	0.92	[62]
	PER in (60–90%) ethanol—results are given for 70% ethanol	-	2.7	[57]
	SE with >99.8% ethanol	5.7	43.5	[42]
Melegueta pepper	Mixing with 95% ethanol	2.0		[63]
	MAC with absolute ethanol	6.16		[64]
	SE with 95% ethanol	12.3		[65]
Javanese pepper	RE with 96% ethanol	12.0		[8]
	SE with 96% ethanol	8.70		[60]
	MAC with 70 and 96% ethanol—results are given for 70% ethanol	14.89		[21]
	SE with 99.5% ethanol	1.00		[45]
	MAE with 96% ethanol	13.94	0.03	[61]
Pink pepper	UAE and SE with >95% ethanol—results are given for UAE	28.6		[55]
	PLE and SE with 99.9% ethanol—results are given for SE	36.00		[9]
	SE, UAE, SEE with 44% ethanol—results are given for UAE	21.00		[19]
Bengali pepper	SE with 90% ethanol	8.6		[66]
	UAE, SE, mixing with ethanol		0.58	[56]
	MAC with 30% ethanol	4.7		[67]

### 2.2. Chemical Composition of Essential Oils

**Figure 1:**
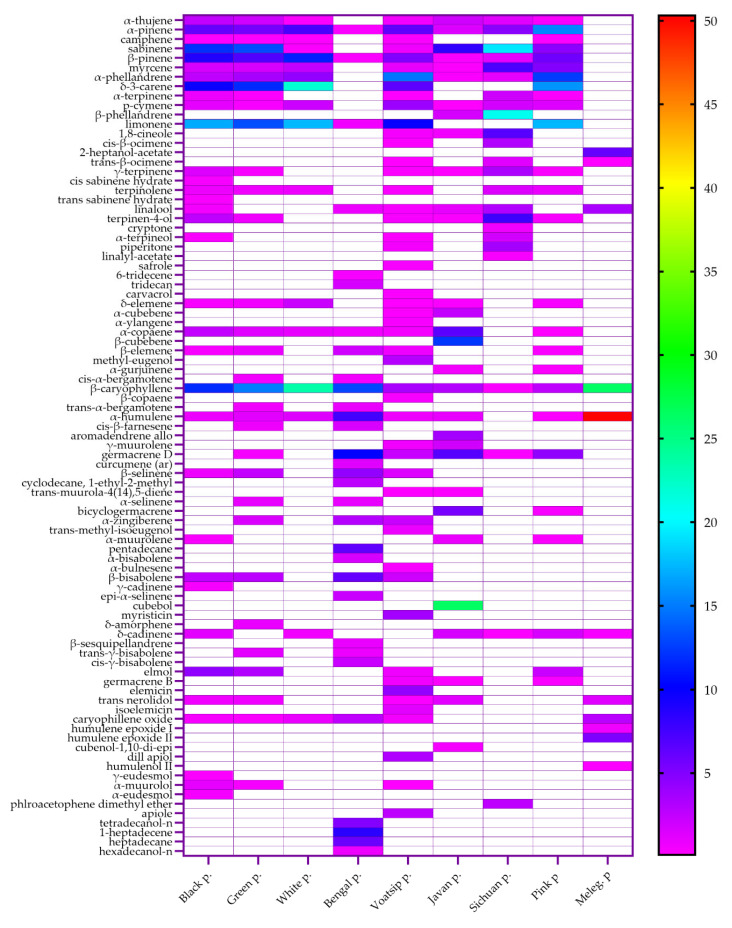
A heatmap of the essential oil constituents and tested species.

The results of the GC-MS analysis of various pepper species’ essential oils are shown in Figure 1 and Table S1 (Supplementary Materials). Black, green, and white pepper belong to the same species, *P. nigrum*, but the fruits are treated in different ways. The essential oils of all three peppers contain large proportions of monoterpenes, of which the following stand out: sabinene (12.01% in black pepper oil and 13.09% in green pepper oil); δ-3-carene (the most abundant in white pepper oil—21.89%); and limonene (present in all three peppers—13.2–17.41%). β-Caryophyllene was the dominant sesquiterpene compound in all three pepper oils (11.76–23.6%). A similar composition was confirmed by other authors [23,37,39,42].

The results obtained for Bengali pepper essential oil revealed the following main compounds: β-caryophyllene (12.86%), germacrene D (10.18%) belonging to sesquiterpenes, and alkene 1-heptadecene (8.47%). Previous studies of the Bengali pepper essential oil have indicated a similar composition, with the dominant components being β-caryophyllene and aliphatic hydrocarbons [23,35,51,52]. Varughese et al. [34] described a sample with β-pinene (43.1%) and α-pinene (15.3%) as the dominant components. Voatsiperifery pepper essential oil had higher amounts of monoterpenes, namely α-pinene (6.39%), β-pinene (5.03%), α-phellandrene (14.77%), δ-3-carene (6.43%), and limonene (9.88%), but significantly lower amounts of β-caryophyllene (3.52%) in comparison to black, green, white, and Bengali pepper. Previous research on Voatsiperifery pepper essential oil is scarce. Weil et al. [29,30] also found high amounts of monoterpenes, with limonene as the most abundant one, while asaricin (13.47%) was the predominant phenyl-propanoid. This component was not identified in our samples.

Two compounds dominated in Javanese or cubeb pepper essential oils—the sesquiterpenes cubebol (26.4%) and β-cubebene (12.32%). Among other compounds, the monoterpene sabinene (8.23%) and sesquiterpenes germacrene D (6.77%) and bicyclogermacrene (5.47%) were present in higher concentrations. Similar amounts of cubebol and β-cubebene were found in a sample from India [45], yet other researchers have reported the lower presence or absence of these compounds [23,40,44,46].

Sichuan pepper (*Z. simulans*) essential oil was characterized by high amounts of the monoterpenes sabinene (19.28%) and β-phellandrene (20.83%), which were present in the highest proportions compared to other pepper essential oils. Myrcene and 1,8-cineole, with 7.17 and 6.75%, were among the most abundant components of the essential oil. The composition of Sichuan pepper essential oil obtained by hydrodistillation has not been described in the literature so far. The results obtained with other extraction methods are not comparable to those for essential oils, and the results obtained in this work provide insights into 24 components of essential oils. A GC-MS analysis of pink pepper essential oil revealed four major monoterpenoid compounds: α-pinene (15.46%), α-phellandrene (12.51%), δ-3-carene (15.98%), and limonene (17.39%). De Oliviera et al. [50] have detected similar content, depending on the peppers’ drying temperatures, while Carneiro et al. [49] reported different results, with smaller content of α-phellandrene, δ-3-carene, and limonene and high amounts of sabinene (31.39%).

Melegueta pepper essential oil was the most specific among all tested samples, revealing only ten identified compounds, of which the two major ones were β-caryophyllene (26.38%) and α-humulene (50.31%). A review of the literature showed that there are pink pepper samples that are also dominated by these two components but with different ratios [26,27,28].

To summarize, the heterogeneity of the pepper essential oil composition can be highlighted. Although they come from the same species, the essential oils of black, green, and white pepper differ in composition, with the essential oil of white pepper differing significantly in composition from those of black and green pepper. Voatsiperifery pepper exhibits the greatest phytochemical diversity, with 49 distinct compounds identified, in contrast to Melegueta pepper, which contains only 11 constituents and over 50% of which is composed of α-humulene.

### 2.3. FTIR and ^1^H NMR Spectra of Essential Oils

**Figure 2:**
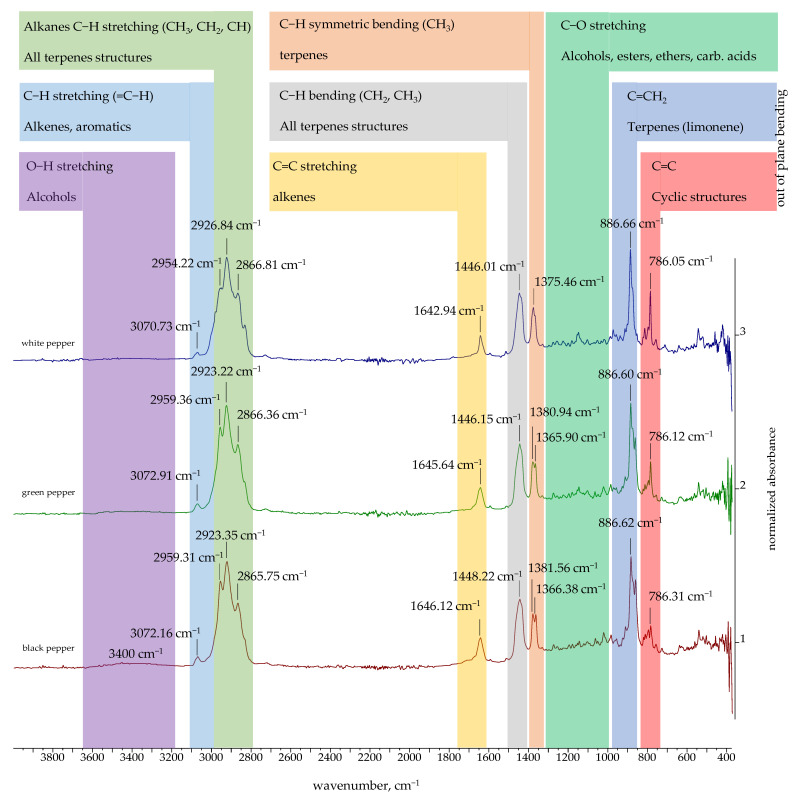
FTIR spectra of *Piper nigrum* species (black, green, and white pepper).

**Figure 3:**
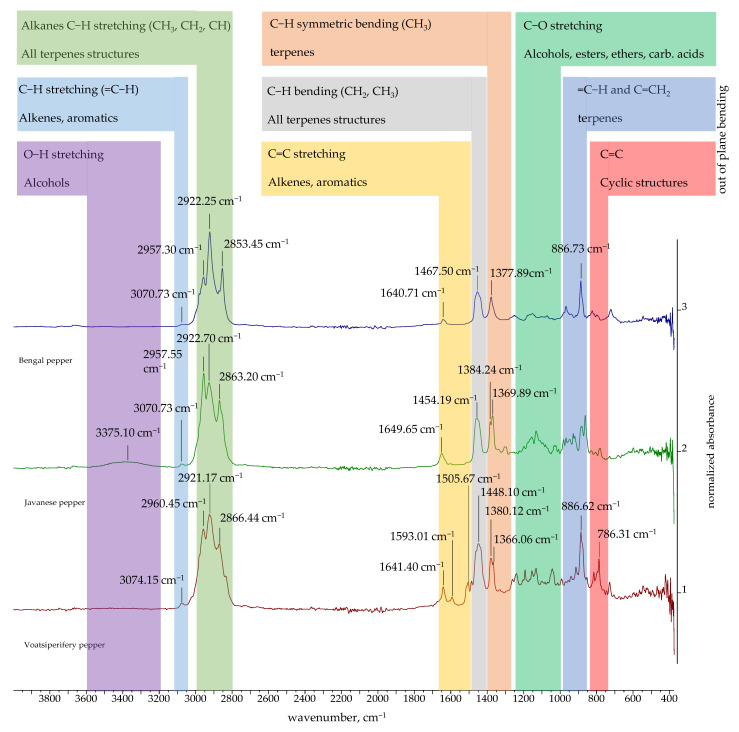
FTIR spectra of Voatsiperifery, Javanese, and Bengal pepper.

**Figure 4:**
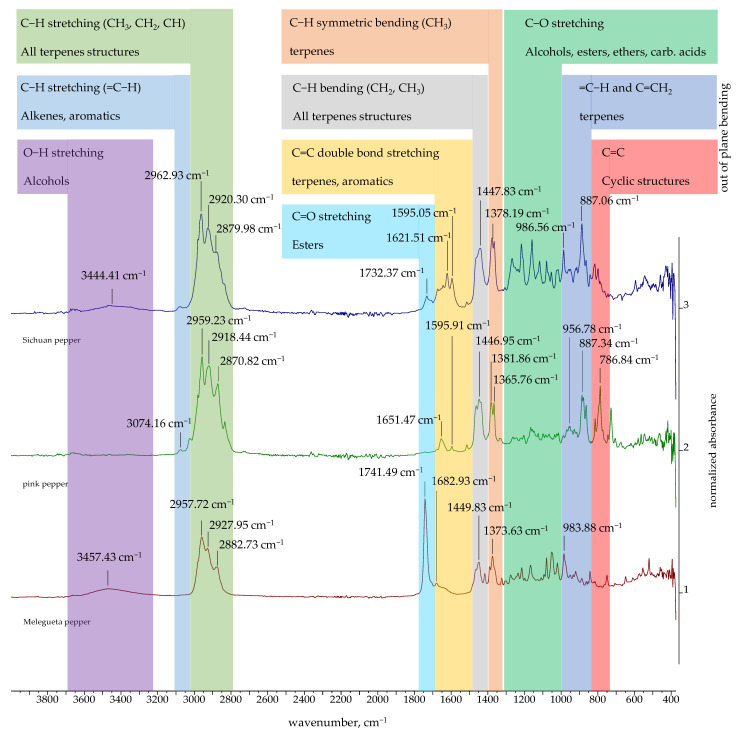
FTIR spectra of Melegueta, pink, and Sichuan pepper.

The FTIR spectra of the EOs obtained in this study are presented in Figure 2, Figure 3 and Figure 4. The FTIR spectra of essential oils enable rapid characterization through the identification of absorption bands specific to particular functional groups. The FTIR spectra of black, green, white, Bengal, and pink pepper indicate that these essential oils are predominantly composed of hydrocarbons. Furthermore, the weak absorption bands related to aromatic C=C stretching indicate the presence of a minor quantity of aromatic chemicals in the pink pepper essential oil. The essential oils of Sichuan and Melegueta pepper exhibit a substantial quantity of oxygenated compounds, which display absorption bands indicative of alcohols, esters, ethers, ketones, and carboxylic acids. The FTIR spectra of Javanese pepper essential oil exhibit absorption bands corresponding to hydrocarbons and oxygenated compounds. The most distinctive FTIR spectra belong to Voatsiperifery pepper essential oil, characterized by absorption bands typical of aromatic compounds.

The absorption bands of the FTIR spectra directly reflect the functional groups of the compounds identified in the essential oils by GC-MS. Several intense peaks (2959.31, 2923.35, 2865.75 cm^−1^) correspond to the aliphatic C-H stretching of the numerous methyl and methylene groups found in all major terpenes and sesquiterpenes present in black pepper essential oil. The peak at 1646.12 cm^−1^ corresponds to C=C double bond stretches, which can be attributed to terpenes like limonene and sabinene. The =C-H stretching peak for the alkene carbon at 3072.16 cm^−1^ supports the high degree of unsaturation in the oil’s composition. Similar results were obtained for green pepper essential oil: C-H stretches of methyl and methylene groups (2959.36, 2923.22, 2866.36 cm^−1^); C=C double bond stretches (1645.64 cm^−1^) and =C-H stretches for the alkene carbon (3072.91 cm^−1^). The major compounds of both essential oils are limonene and sabinene, so similarities in their FTIR spectra were expected. White pepper essential oil is characterized by a high concentration of δ-3-carene. The peak that can be attributed to δ-3-carene and other terpenes with double bonds is a key absorption band for C=C double bond stretches at 1642.94 cm^−1^. A low-intensity broad signal at around 3400 cm^−1^ in the FTIR spectra of black and green pepper corresponds to alcohols detected using GC-MS. Black and green pepper essential oils contain 10.54% (linalool, terpinen-4-ol, α-terpineol, elemol, *trans* enorlidol, γ- and α-eudesmol, α-muurolol) and 4.99% (terpinen-4-ol, elemol, *trans* enorlidol, α-muurolol) alcohols, respectively.

Besides aliphatic C-H stretches (2957.72 and 2882 cm^−1^), the FTIR spectrum of Melegueta pepper is characterized by a carbonyl C=O stretch (1741.49 cm^−1^) due to the presence of esters like 2-heptanol acetate and C-O stretches (1235.24 and 1214.99 cm^−1^) that can be attributed to oxygenated compounds. The FTIR spectrum of Voatsiperifery pepper is characterized by aliphatic C-H stretches (2982.06, 2960.45, 2921.17, and 2866.40 cm^−1^)—a clear signature of methylene and methyl groups—and alkene C=C stretches (1641.40 cm^−1^), which are consistent with the high concentrations of terpenes (limonene and β-phellandrene), as well as C=C stretching within an aromatic ring (1593.01, 1505.67 cm^−1^), which proves the presence of minor aromatic compounds identified by GC-MS, such as cymenes, apiole, dill apiole, elemicin, and myristicin. Besides aliphatic C-H stretches (2863–2981 cm^−1^) and alkene C=C stretches (1649.65 cm^−1^), in the FTIR spectrum of Javanese pepper essential oil, a hydroxy O-H stretch at 3375.10 cm^−1^ can be observed. The presence of this absorption band suggests that the oil contains minor amounts of alcohols, such as linalool or terpinen-4-ol, which were also identified by GC-MS.

The pink pepper essential oil’s FTIR spectrum is characterized by the aliphatic C-H stretches (2833–2981 cm^−1^) of the methyl and methylene groups in monoterpenes and alkene C=C stretches (1651.47 cm^−1^) that can be attributed to monoterpenes containing double bonds, like limonene and pinenes, as well as C=C stretching within the aromatic rings of, for instance, para-cymene. The absorption bands of Bengali pepper essential oil confirm the presence of hydrocarbons, monoterpenes, and sesquiterpenes like β-caryophyllene, as well as β-pinene (aliphatic C-H stretches: 2853–2981 cm^−1^) and terpenes with double bonds like β-caryophyllene, β-pinene, and limonene (alkene C=C stretches: 1640.71 cm^−1^). The lack of a broad peak around 3300–3600 cm^−1^ is a key observation. While GC-MS identifies the alcohol (E)-nerolidol, its concentration or the presence of other components may cause its hydroxyl group’s absorption band to be either very weak or absent. The FTIR spectrum of Sichuan pepper essential oil confirms presence of the following major compounds: terpinen-4-ol (hydroxyl O-H stretch at 3444.41 cm^−1^), piperitone (ketone carbonyl C=O stretch at 1732.37 cm^−1^), and monoterpenes (sabinene, pinenes, and cimene) (alkene C=C stretch).

The FTIR spectra of the essential oils of black pepper [68] and Melegueta pepper [69] have already been published. Black pepper essential oil’s FTIR spectrum aligns with the one previously published [68], as both oils’ chemical compositions are comparable. The FTIR spectra of the Melegueta pepper essential oil produced in this research and in a previously published work [69] differ due to their different chemical compositions.

**Figure 5:**
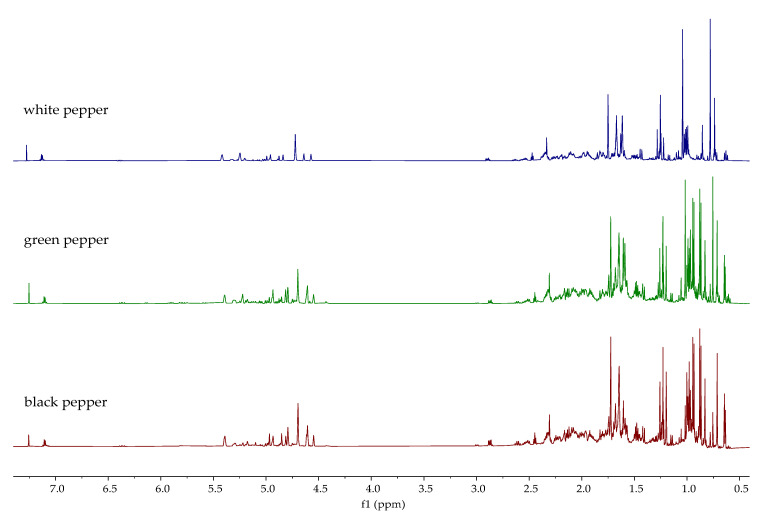
^1^H NMR spectra of *Piper nigrum* species (black, green, and white pepper).

**Figure 6:**
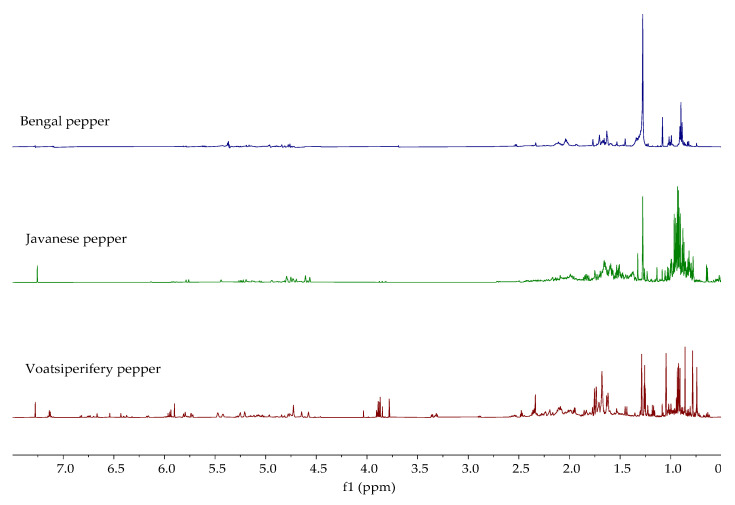
^1^H NMR spectra of Voatsiperifery, Javanese, and Bengal pepper.

**Figure 7:**
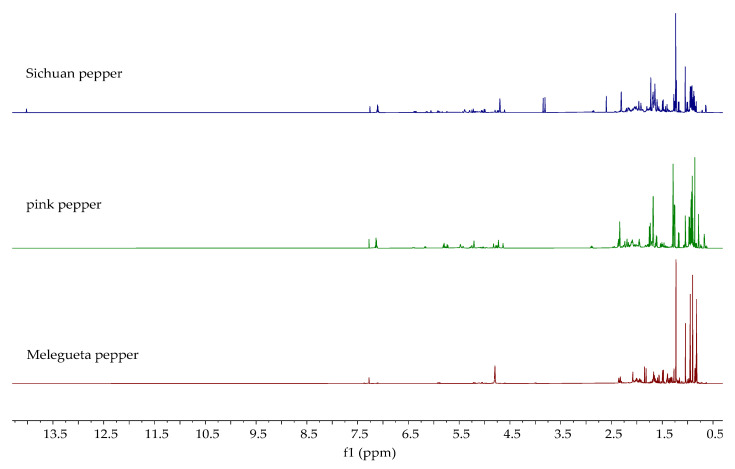
^1^H NMR spectra of Melegueta, pink, and Sichuan pepper.

The ^1^H NMR spectra of the analyzed essential oils are shown in Figure 5, Figure 6 and Figure 7. The ^1^H NMR spectra of all investigated oils provide definitive structural evidence for the compounds identified by GC-MS and FTIR. Unlike essential oils that contain a significant proportion of the main constituent (e.g., lemon peel, bay leaf, clove, star anise, oregano) [31], which can be identified with certainty by ^1^H-NMR, no single constituent stands out as the main constituent in the essential oils analyzed in this study. Due to the complexity of the samples, only the presence of certain types of compounds, such as terpenes, alcohols, esters, and fatty acids, can be confirmed. As can be seen from the observed spectra, aliphatic protons (0–3.0 ppm) dominate in all samples, mainly due to the presence of methylene protons of saturated and unsaturated fatty acids and terpenes such as α- and β-pinene in white pepper, which were also identified by GC-MS. Some additional similarities in the ^1^H NMR spectra between *P. nigrum* species can be observed. The complex pattern of the signals in the alkene proton range (δ 4.5–6.5 ppm) is clear evidence of the protons on the double bonds, which indicate the presence of compounds like limonene, sabinene, and β-caryophyllene in black and green pepper and δ-3-carene, myrcene, and limonene in white pepper. The presence of minor peaks in the aromatic proton region (δ > 6.5 ppm) is definitive evidence for the presence of the aromatic ring protons of o- and p-cymene and other related compounds found in the GC-MS analysis. Regarding Melegueta pepper essential oil, apart from terpenes and fatty acids, the presence of an ester group at 4.01 ppm confirms the presence of 2-heptanol acetate. Besides aliphatic, alkene, and aromatic protons, two singlets can be observed at 3.81 ppm and 3.85 ppm in the ^1^H NMR spectrum of Sichuan pepper essential oil. The singlets correspond to methoxy groups (-OCH_3_). The presence of methoxy groups most likely originates from phloroacetophenone 2,4-dimethyleter, identified by GC-MS. The spectra of the Voatsiperifery, Javanese, and Bengal pepper oils appear similar to those of the oils displayed in Figure 5 and Figure 7 and the NMR spectra from previously published studies on black pepper oils [68]. As can be seen, the Voatsiperifery pepper essential oil’s spectrum reveals its unique chemical profile as a terpene-rich sample with notable aromatic components. In the ^1^H NMR spectrum of Javanese pepper essential oil, signals in the alkene proton range can be attributed to major alkene-containing sesquiterpenes like β-copaene and germacrene D. The presence of a hydroxyl proton in the spectra of pink pepper and Bengal pepper essential oils at 3.69 ppm is consistent with the identification of E-nerolidol by GC-MS.

The ^1^H NMR spectra of black pepper [68], Melegueta pepper [69], and pink pepper [70] essential oils can be found in the available literature. Thanh-Tam Huynh et al. [68] investigated the influence of the extraction method (conventional and microwave-assisted hydrodistillation, supercritical CO_2_ extraction, and solvent extraction) of the essential oil from black pepper on the chemical composition and antioxidant and antibacterial properties of the obtained EOs. The ^1^H NMR spectra of the EOs obtained by both hydrodistillation methods are in concordance with those of the black pepper EO obtained in this study. Chemical shifts are grouped into two regions, 0.2–3.0 ppm and 4.6–5.4 ppm, which correspond to signals characteristic of the major EO compounds (b-caryophyllene, 3-carene, D-limonene). A group of authors identified and described the quantification of the major primary and secondary metabolites in both black and white pepper by using the ^1^H, COSY, HSQC, and HMBC NMR techniques [71]. Rivera-Perez, for the first time, combined ^1^H NMR spectroscopy with chemometrics methods (principal component analysis (PCA) and orthogonal partial least squares discriminant analysis (OPLS-DA)) for the discrimination of black pepper samples based on their geographical origin—i.e., Brazil, Vietnam, or Sri Lanka—and processing quality (sterilized or non-sterilized spice) [72]. Be et al. investigated the effects of coatings incorporated with black pepper essential oil (CIBPEO) on the taste of Jinhua ham after 4 months of storage by using ^1^H NMR and multivariate data analysis [73]. Angaye and Inengite [69] studied the spectral and antimicrobial properties of Melegueta pepper essential oil. The significant differences between the ^1^H NMR spectra of their EO and the EO obtained in this study imply different chemical compositions of Melegueta essential oils. Cerceau et al. [70] developed and validated a method for the quantification of α-pinene in various essential oils. This group of authors did not provide the chemical composition of the essential oil. However, variations in the ^1^H NMR spectra suggest the presence of different compounds in such essential oils.

### 2.4. Piperine Content in Ethanolic Extracts

#### 2.4.1. Determination of Piperine Content by HPLC

Piperine was solely identified in five pepper species (black, green, white, Bengali, and Voatsiperifery) using the HPLC method. The data obtained are shown in Table 4 and compared with the existing literature. The measured piperine content in the ethanolic extracts of black and green pepper was slightly lower than those reported in the literature. The piperine content obtained is impacted by both the origin and the extraction procedure, as well as the ethanol purity, ranging from 70% to 96%. This complicates the comparison of the data obtained with the existing literature. No literature exists regarding ethanol extracts of Voatsiperifery pepper. The literature indicates that ethanolic extracts of Javanese pepper also contain piperine. To ascertain the presence of piperine in the extracts, their FTIR and ^1^H NMR spectra were recorded and compared with the spectra of piperine.

#### 2.4.2. Verification of Piperine Presence in Ethanolic Extracts by FTIR

**Figure 8:**
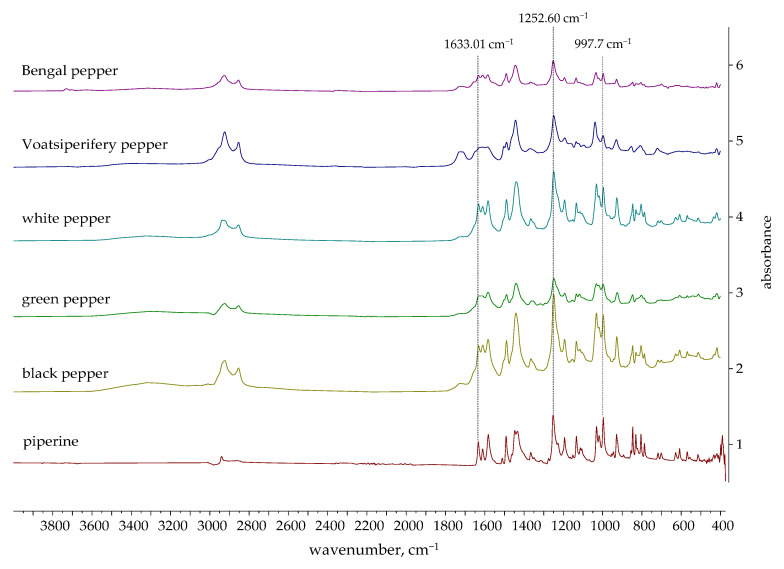
FTIR spectra of piperine and ethanolic extracts of various *Piper* species, showing characteristic signals for piperine.

**Figure 9:**
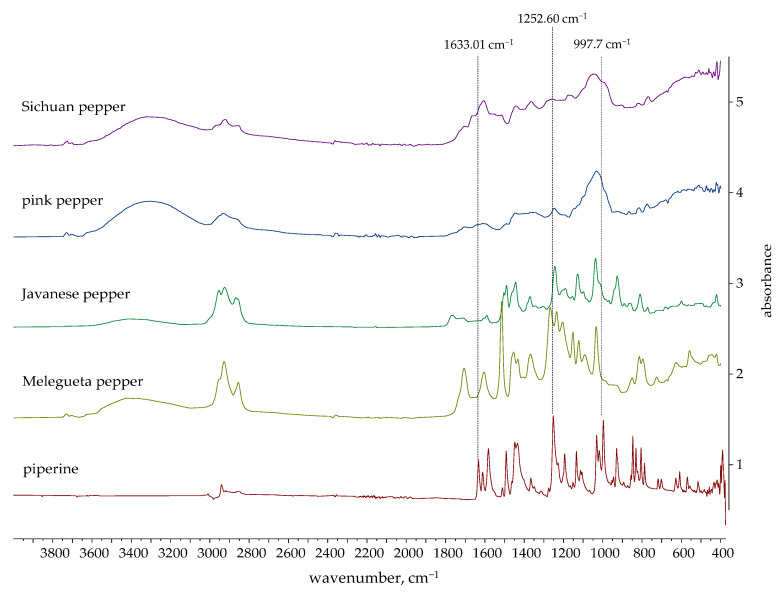
FTIR spectra of piperine and ethanolic extracts of pepper species lacking piperine.

The FTIR spectra of piperine and the ethanolic extracts are shown in Figure 8 and Figure 9. Figure 8 shows the spectra of extracts in which the presence of piperine was confirmed, while Figure 9 shows the spectra of extracts in which the characteristic peaks of piperine were not observed.

Several signals are particularly characteristic of the piperine molecular structure [76,77,78]:

- 1633.01 cm^−1^: A very strong band that is highly characteristic of conjugated C=O stretching (amide I band) from the amide functional group in piperine;
- 1252.60 cm^−1^: A strong absorption band characteristic of the asymmetric stretching of the C-O-C bonds within the methylenedioxy group, a unique structural feature of piperine;
- 997.07 cm^−1^: A strong and sharp peak characteristic of the out-of-plane bending of the trans C=C double bond in piperine’s alkene side chain.

The spectra of black, green, white, Voatsiperifery, and Bengal pepper distinctly exhibit peaks characteristic of piperine, supporting the HPLC results. Other supporting peaks, such as aliphatic C-H stretches (~2959 cm^−1^), aromatic C=C stretches (~1582 cm^−1^, ~1489 cm^−1^), and various C-O-C stretches (~1193, ~1133, ~1031 cm^−1^), are also consistent with the piperine structure.

FTIR spectroscopy confirmed the presence of piperine determined by HPLC, so it can be concluded that FTIR is a potential technique of choice for monitoring the marker compound piperine in the extraction process from pepper. Future research will be focused on developing an FTIR method for the quantitative analysis of the main bioactive marker, piperine, in the studied pepper species.

#### 2.4.3. Verification of Piperine Presence in Ethanolic Extracts by ^1^H NMR

**Figure 10:**
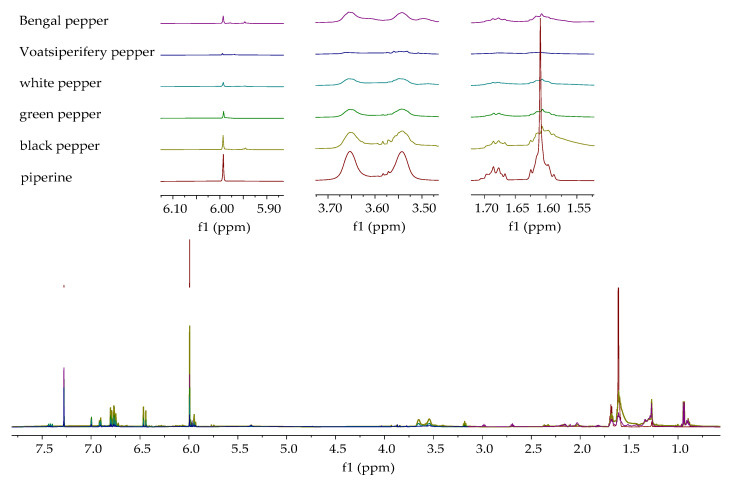
^1^H NMR spectra of piperine and ethanolic extracts of various *Piper* species, showing characteristic signals for piperine.

**Figure 11:**
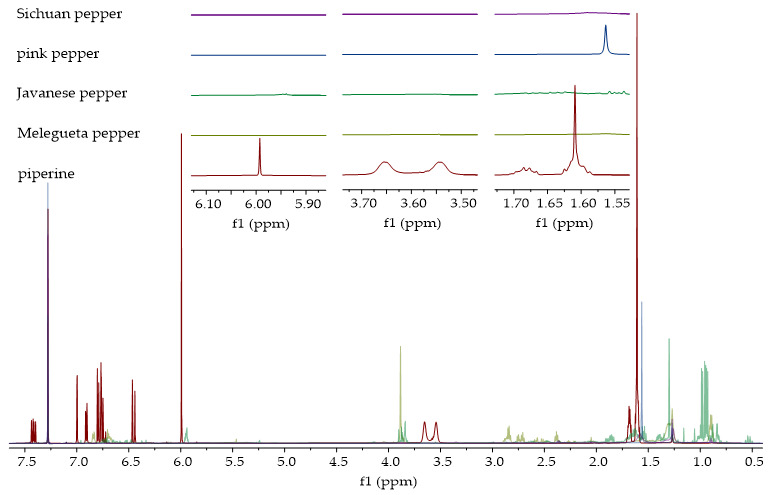
^1^H NMR spectra of piperine and ethanolic extracts of pepper species lacking piperine.

The ^1^H NMR spectra of piperine and the ethanolic extracts of the analyzed pepper species are shown in Figure 10 and Figure 11. Figure 10 displays the spectra of extracts in which the presence of piperine was confirmed, while Figure 11 shows the spectra of extracts in which the characteristic signals of piperine were not observed. Characteristic signals for piperine [43] can be observed in the ^1^H NMR spectra of the ethanolic extracts of black, green, white, Voatsiperifery, and Bengal pepper (Figure 10), matching the HPLC findings. One of the most prominent signals is a sharp singlet around 6.0 ppm, which belongs to the two protons on the methylene group (CH_2_) of the methylenedioxy bridge. The protons of the piperidine ring appeared in the aliphatic region between 1.5 and 1.7 ppm. The two protons on the carbon adjacent to the nitrogen are typically shifted downfield, appearing between 3.5 and 3.7 ppm. Signals characteristic of piperine were not identified in the spectra of Sichuan, pink, Javanese, and Melegueta pepper (Figure 11).

### 2.5. Antioxidant Activity of EOs and EEs

#### 2.5.1. Total Phenolic and Flavonoid Content in EOs and EEs

The total phenolic (TPC) and flavonoid content (TFC) in the EOs and EEs of the investigated pepper species is presented in Table 5. GC-MS analysis detected only one phenolic compound, carvacrol, in the essential oil of Voatsiperifery pepper. A minor amount of phenolic compounds was identified in all essential oils, suggesting that some phenolic compounds may have been volatilized with the steam during hydrodistillation and consequently not detected by GC-MS analysis. Specifically, GC-MS analysis identified between 90.45% (Voatsiperifery pepper) and 97.73% (white pepper) of the components, allowing for the presence of phenolic compounds. In addition, the FC reagent may have reacted with some non-phenolic compounds, resulting in overestimated values [79]. For all pepper species, the essential oils contained lower amounts of phenolic compounds than their corresponding ethanolic extracts. Phenolic compounds and flavonoids are generally large, polar molecules that are non-volatile and are not efficiently carried over by hydrodistillation. Ethanol, as a polar solvent, is highly effective in dissolving polar non-volatile compounds, which are rich in phenolics and flavonoids. The absence or minimal quantities of phenolic compounds and flavonoids in essential oils result in the superior antioxidant activity of ethanolic extracts. Sichuan pepper essential oil and Melegueta pepper ethanolic extract contain the highest amounts of phenolic compounds, while Melegueta pepper ethanolic extracts contain the highest amounts of flavonoids. For the *Piper nigrum* EOs, the TPC decreases in the following order: black pepper > green pepper > white pepper. The main reasons for this trend are processing differences between them [25,38,42,80]. Black pepper is harvested from fully grown yet unripe pepper berries and then dried. The entire pericarp, a significant source of phenolic and flavonoid compounds, is retained. Green pepper is harvested from unripe berries and preserved without drying or removing the outer skin. White pepper is produced from fully ripe berries that have their outer skin removed through a process of retting (soaking) and washing. The removal of the pericarp reduces the TPC compared to black pepper. However, due to the likely differing geographic origins, it is challenging to justify this order.

Table 6 presents a summary of the literature data regarding the total phenolic (TPC) and flavonoid (TFC) content of essential oils and ethanol extracts for the peppers studied in this research.

Most of the results obtained in this study are difficult to compare with those in the literature since they are not given in the same units. This is especially true for the TFC, which is often expressed as quercetin [21], catechin [59], catechol [60], or gallic acid [20] equivalents. The comparison regarding TPC is approximate, as most published results are expressed in mg GAE/g. There are no data in the available literature for the following essential oils and ethanolic extracts:

- Essential oils: TPC for Javanese, pink, Bengal, and Sichuan pepper;
- Ethanolic extracts: TPC and TFC for white, Voatsiperifery, and Sichuan pepper.

Significant variations in antioxidant activity are evident among the essential oils and ethanolic extracts derived from peppers of diverse origins and produced through various procedures. For example, the TPC of black pepper essential oil extracted via hydrodistillation ranged from 1.21 mg GAE/g (India) [24] to 237.556 mg GAE/g (Hainan Province, China) [80], demonstrating the impact of the geographical origin. The impact of the extraction method on the antioxidant activity of ethanolic extracts was extensively investigated in several studies [5,6,9,19,21]. Milenković et al. [5] examined the impact of the extraction method and process conditions on the antioxidant activity of black pepper ethanolic extracts. The maximum TPC was obtained using UAE and RE at a boiling temperature. Zhang et al. [6] examined the impact of the extraction method on the quality of green pepper ethanolic extracts. Although the maximum oleoresin production was obtained using UAE, the oleoresin produced with MAE had superior total phenolic content. Andrade et al. [19] investigated various extraction methods and solvents to obtain ethanolic extracts of pink pepper. The optimal yield and antioxidative activity of oleoresin were obtained via SE with ethanol. The TPC exhibited a marginal increase with the application of ethyl acetate. Dias et al. [9] reported that ethanolic extracts of pink pepper obtained through PLE demonstrated higher total phenolic content compared to those obtained via SE. The results obtained by De Mello et al. [55] demonstrate that SE is a more efficient method than UAE in extracting the TPC and TFC from pink pepper ethanolic extracts. The influence of the ethanol concentration on the TPC and TFC of ethanolic extracts from Javanese pepper has been examined by Dwita et al. [21]. The extract produced with 70% ethanol exhibited higher TPC, while the extract obtained with 96% ethanol demonstrated greater TFC. These findings suggest that optimizing the ethanol concentration is crucial in enhancing the yields of bioactive compounds in pepper extracts. Future research could explore the synergistic effects of varying extraction conditions to further improve the efficiency of these methods. In addition, the identification of flavonoids in ethanolic extracts by means of HPLC/MS, for instance, would provide additional knowledge about the chemical composition and better insights into the antioxidant activity.

Except for the Melegueta pepper essential oil, the reported values for the TPC are generally higher than those obtained in this study. Zhang et al. [80] have reported significantly higher TPC (237.556 mgGAE/g) for black pepper essential oil. The TPC values of black and green pepper essential oils [24], as well as Voatsiperifery pepper essential oil [29], are comparable to those reported in prior studies. The essential oil of Melegueta pepper obtained by Adefegha et al. [82] contained high amounts of eugenol (82.2%), and the reported TPC value was 5.45 mg GAE/L. The TPC for Melegueta pepper essential oil (Table 4) equals 294.93 mg GAE/L, which is significantly higher. As mentioned previously, the wide range of TPC and TFC values for the reported ethanolic extracts can be attributed to the different extraction methods, process conditions, and ethanol purities used. The TPC for black pepper EEs ranges from 5.89 (mixing with 80% ethanol) [59] to 89.90 (Soxhlet with 96% ethanol) [8] mg GAE/g. The TFC for black pepper EEs ranges from 17.2 (Soxhlet with 96% ethanol) [8] to 73.15 (ultrasonic extraction with 70% ethanol) [5] mg RE/g. In the same work, using the same conditions as in this study (ultrasonic extraction with 70% ethanol), the obtained value for TPC was 85.64 mg GAE/g. For the green pepper EE, the TPC ranges from 3.75 (ultrasonic extraction with absolute ethanol) [6] to 14.15 mg GAE/g (mixing with 80% ethanol) [59]. Both reported values are significantly lower than those obtained in this study. The published TPC values for Javanese pepper range from 75.60 (Soxhlet with 96% ethanol) [5] to 206.99 mg GAE/g (maceration with 70% ethanol) [21]. Only one value for TFC was published in the form of rutin equivalents (4.5 mg RE/g) (Soxhlet with 96% ethanol) [8]. For a pink pepper EE obtained by ultrasonic extraction with absolute ethanol, the TPC was 14.2 mg GAE/g, which was lower than for an extract obtained by Soxhlet extraction (TPC = 60 mg GAE/g) [19].

#### 2.5.2. Radical Scavenging Activity of EOs and EEs

The antioxidant activity of essential oils and ethanolic extracts of different pepper species was evaluated using the DPPH method. The results are presented in Table 7. The EC_50_ results indicate that ethanol extracts exhibit considerably greater antioxidant activity compared to the corresponding essential oils. The Sichuan pepper essential oil and pink pepper ethanol extract have the highest antioxidant activity. The extraction techniques for essential oils and ethanol extracts yield distinctly diverse molecules; hence, differing antioxidant activity is anticipated. Essential oils mostly consist of terpenes. Black, green, white, Sichuan, and pink pepper are primarily composed of monoterpenes, while Bengal, Javanese, and Melegueta are predominantly composed of sesquiterpenes. Despite monoterpenes exhibiting superior antioxidant activity compared to sesquiterpenes [86], the corresponding quantity of individual terpenes is not essential for the total antioxidant activity [87]. The antioxidant activity of some essential oils analyzed in this study can be explained by their chemical compositions and TPC values. Sichuan pepper essential oil possesses the highest concentrations of monoterpenes, monoterpene alcohols, and total phenols, which accounts for its superior antioxidant activity. White pepper essential oil exhibits the poorest antioxidant activity, with no terpene alcohols found and a low TPC value. Aside from these two essential oils, the ordering can also be justified for Melegueta pepper essential oil, which ranks seventh.

Wang et al. [87] examined the antioxidant properties of essential oils from black and white pepper, as well as standards including α- and β-pinene, caryophyllene, limonene, linalool, and 2- and 3-carene. The essential oils exhibited superior antioxidant activity compared to the standards. The EC_50_ values obtained by the DPPH assay for all standards were similar.

The EC_50_ values indicate that Sichuan, pink, Bengal, and Voatsiperifery essential oils have significantly greater antioxidant activity than the other essential oils analyzed in this study. Sichuan pepper essential oil contains a substantial quantity of oxygenated compounds, notably monoterpene alcohols like terpinen-4-ol and linalool, which have antioxidant activity [86,88,89,90]. The synergistic effect of the essential oil constituents is further demonstrated by the similar antioxidant activity of Bengal pepper and pink pepper essential oils. Sesquiterpenes define the former (Bengal), while monoterpenes define the latter. The essential oil of Bengal pepper has a small quantity of terpene alcohols and oxides but significant concentrations of antioxidant-active compounds (β-caryophyllene, α-humulene, and germacrene D) [90,91]. Pink pepper essential oil consists of a significant quantity of monoterpenes with antioxidant properties, including α- and β-pinene, limonene, and myrcene [86,90,91]. Voatsiperifery pepper essential oil is characterized by its high concentrations of aromatic compounds, several of which possess antioxidant properties, including methyl eugenol, methyl isoeugenol, p-cymene, elemicin, isoelemicin, dillapiol, safrole, and myristicin [90,92,93,94,95,96,97]. The essential oil of Melegueta pepper, although rich in oxygenated components, is predominantly composed of sesquiterpenes, particularly β-caryophyllene and α-humulene. Both compounds have antioxidant properties [90]; however, their limited antioxidant efficacy can be ascribed to the antagonistic effects of the oil constituents.

Table 8 presents an overview of the published EC_50_ values of the essential oils and ethanolic extracts of the peppers examined in this study. The antioxidant activity of the essential oils extracted in this study is lower than that reported in previously published data for all peppers examined. The majority of essential oils are obtained through Clevenger hydrodistillation; thus, the discrepancies between the published values and those reported in this article arise from variations in origin and chemical composition. The extraction method, ethanol concentration, and process conditions significantly influence the chemical composition of the extracts, altering their antioxidant activity. This variability complicates the comparison of the data from this study with previously published results. Milenković et al. [5] demonstrated that the extraction method affects the antioxidative activity of ethanolic extracts of black pepper. Extracts obtained through RE and SE exhibited the highest antioxidative activity. Zhang et al. [6] reported that, among the extraction methods tested, green pepper oleoresin obtained via MAC exhibited the highest antioxidative activity, as assessed by the DPPH and ABTS assays. Andrade et al. [19] investigated the effects of the extraction methods on the antioxidative activity of pink pepper oleoresin, finding that the highest antioxidative activity was obtained through SE using ethanol. These findings point out the importance of selecting appropriate extraction methods to maximize the antioxidative potential of various pepper extracts. Future research could focus on optimizing these techniques further or exploring additional pepper varieties to uncover even more potent antioxidative compounds.

The antioxidant effect of pepper may be associated with other biological effects, such as anti-inflammatory, cytoprotective, anti-aging, and others. The components that contribute to the antioxidant effects of ethanolic extracts usually have polyphenolic structures. For *P. nigrum*, as the most researched pepper, the presence of caffeic, chlorogenic, and gentisic acids, as well as flavonoids with apigenin and kaempferol as aglycones, has been proven [100,101,102]. Many phenolic compounds have been recorded in Bengali pepper, among which rutin and chicoric and ferulic acids are strong antioxidants [103]. Javanese pepper is characterized by lignans (cubebin), polyphenolic compounds that also contribute to the antioxidant potential of this species, while pink pepper is rich in anthocyanin derivatives, such as cyanidin and pelargonidin, both strong antioxidants [1]. Gingerol and paradol, phenolic compounds from Melegueta pepper, could be responsible for its antiradical activity [104]. There is no relevant polyphenol compositional data for Voatsiperifery and Sichuan pepper.

It is evident from the results of this study that Sichuan pepper essential oil showed the best antiradical activity (EC_50_ = 7.35 mg/mL) and was also the richest in phenols. Among the ethanolic extracts, Melegueta pepper stood out (EC_50_ = 96.33 μg/mL), containing the highest levels of phenols and flavonoids. Additionally, a strong correlation was found between the total phenolic content and EC_50_ for both essential oils and ethanolic extracts (r = −0.7124 and −0.8979, respectively) and between the total flavonoid content and EC_50_ of ethanolic extracts (r = −0.7313). These results indicate a significant contribution of phenolic constituents to the observed antiradical effects. Although the presence of polyphenolic compounds in pepper fruits has been reported, studies focusing on their quantification and their contributions to the antioxidant activity remain limited. This gap underscores the necessity of further research aimed at accurately determining the concentrations of key polyphenolic constituents in peppers. Future research should aim to achieve the more comprehensive characterization of polyphenolic compounds and to optimize the extraction methodologies to maximize the biomedical potential of the resulting extracts.

### 2.6. Antimicrobial Activity of Essential Oils and Ethanolic Extracts

This study investigated the antimicrobial potential of nine pepper-derived essential oils and solvent extracts—specifically black pepper, green pepper, white pepper, Melegueta pepper, Voatsiperifery pepper, Javanese pepper, pink pepper, Bengali pepper, and Sichuan pepper—against five clinically relevant bacterial strains: *Escherichia coli*, *Enterococcus faecalis*, *Klebsiella pneumoniae*, *Pseudomonas aeruginosa*, and *Staphylococcus aureus*. These essential oils were evaluated against ceftazidime (CAZ) and ciprofloxacin (CIP), two common antibiotics. Previous studies have predominantly focused on the antibacterial activity of black pepper, given its widespread use. However, research on other pepper varieties, including their oils and extracts, remains limited, thereby underscoring the scientific contribution of this study. The results are represented in Table 9 and Table 10.

All tested samples exhibited measurable inhibitory activity, with complete growth inhibition observed at 512 µg/mL. Solvent extracts of Javanese pepper, pink pepper, Bengali pepper, and Sichuan pepper had notably stronger antibacterial effects compared to their corresponding essential oils. This enhanced activity can be attributed to the enrichment of polar secondary metabolites [10], including phenolic acids, flavonoids, and alkaloids, which are more efficiently solubilized in polar extraction solvents [105,106] than in hydrophobic essential oil fractions dominated by volatile monoterpenes and sesquiterpenes. Such polar constituents exhibit superior membrane permeability and enhanced interactions with intracellular targets, resulting in greater antimicrobial potency.

The higher inhibitory efficacy of Javanese, pink, Bengali, and Sichuan pepper extracts is consistent with their distinct phytochemical profiles. These species contain elevated levels of alkaloid derivatives, terpenoids, and phenolic compounds, known to exert multiple antimicrobial mechanisms, such as the disruption of bacterial cytoplasmic membranes, inhibition of key metabolic enzymes, and interference with nucleic acid synthesis. For example, Sichuan pepper is rich in alkylamides (e.g., hydroxy-α-sanshool) with well-documented antimicrobial properties, while Javanese and Bengali peppers contain diverse piperine analogs with broad-spectrum bacteriostatic effects. Pink pepper, although belonging to a different taxonomic lineage, provides synergistic antimicrobial action through a combination of monoterpenes, sesquiterpenes, and phenolic constituents. Conversely, black, green, and white pepper (*Piper nigrum*) are characterized by relatively uniform phytochemical profiles dominated by piperine and terpenoids [107], which confer antimicrobial activity but with a narrower spectrum and reduced potency. Melegueta and Voatsiperifery peppers, while bioactive, appear to contain comparatively lower concentrations of highly potent constituents.

With respect to bacterial susceptibility, *E. faecalis* exhibited the lowest sensitivity across all tested samples. The reduced susceptibility of *E. faecalis* to essential oils relative to *S. aureus* is best explained by species-intrinsic resistance mechanisms. In *E. faecalis*, multidrug efflux systems, biofilm formation, and adaptive stress responses collectively lower intracellular exposure and mitigate the activity of hydrophobic essential oil components. Although both organisms are Gram-positive and lack an outer membrane, differences in the cell envelope architecture and membrane hydrophobicity influence compound partitioning, permeability, and target engagement. These factors could have led to the consistently higher tolerance profile for *E. faecalis* under the tested conditions. Interestingly, *E. coli*, despite being Gram-negative, was also strongly inhibited, which can be ascribed to the ability of the polar compounds in the extracts to disrupt the integrity of its outer membrane and interfere with intracellular metabolic pathways. According to the literature [108], essential oils are hydrophobic, which enables them to partition into the lipids of the bacterial cell membrane, disturbing the cell structure, rendering them more permeable, and leading to lysis and the leakage of intracellular compounds. At lower concentrations, the Javanese pepper extract displayed the strongest inhibition, with complete growth inhibition at 8 µg/mL against both *E. coli* and *S. aureus*. The Bengali pepper extract was particularly effective against *E. coli*, while the Sichuan pepper extract exhibited pronounced inhibitory activity against *E. coli* and *P. aeruginosa*. Collectively, these results confirm the strong inhibitory effects of these extracts, particularly against *E. coli*, which is in accordance with the literature [109]. In contrast, essential oils and extracts derived from black pepper, green pepper, white pepper, Melegueta pepper, and Voatsiperifery pepper demonstrated their most pronounced activity at 64 µg/mL against *S. aureus*, with minimal inhibition of *E. faecalis*.

In summary, pepper-derived essential oils and solvent extracts exhibit broad-spectrum antimicrobial activity, with extracts displaying consistently higher efficacy than oils due to their richer content of polar secondary metabolites. The observed variability in bacterial susceptibility reflects the influence of structural and physiological differences in bacterial cell envelopes, underscoring the importance of both the phytochemical composition and microbial resistance mechanisms in determining the effectiveness of natural antimicrobial agents.

### 2.7. Trace Element Content

Micronutrients, or trace elements, are essential for the body in amounts typically less than 100 mg per day. Despite being needed in small amounts, they are important for metabolism, hormone regulation, enzyme function, and many other biological processes. The content of trace elements in plant material is determined by the presence of minerals in the environment in which the plant grows and the ability of the plant to absorb them. Mineral nutrients from the soil reach plants by mechanisms such as root interception, the mass flux of nutrients dissolved in or carried by soil water, and diffusion within the soil [110,111]. Certain microelements have antioxidant and anti-inflammatory properties, which is why they are a key factor in maintaining redox homeostasis and, thus, in preventing oxidative stress. Copper, iron, manganese, selenium, and zinc act as important cofactors of the antioxidant enzymes catalase, glutathione peroxidase, and superoxide dismutase [112].

In this study, the content of microelements in dried plant material from different pepper varieties was determined using total reflection X-ray fluorescence (TXRF). TXRF offers several advantages over other spectroscopic techniques, such as the small amount of sample required, the simultaneous determination of multiple elements, reduced matrix effects, and a simpler quantification approach over a wide dynamic range due to internal standardization. In addition, low-power TXRF benchtop systems are cost-effective, as they do not require gas or cooling media. This has promoted the use of such systems for the determination of trace elements in many different field applications. To our knowledge, this is the first TXRF-based multielement profile described for the studied pepper varieties. The results are presented in Table 11.

The profiles were species-specific. The levels of microelements varied significantly in the pepper samples depending on the species. In this study, the values determined for manganese ranged from 14.62 mg/kg to 136.61 mg/kg. Melegueta and Voatsiperifery peppers were the most enriched in manganese. The permissible limit for Mn in edible plants set by the FAO/WHO (1984) is 2 mg/kg [113]. The results show that all plants can accumulate manganese above this limit. Although Mn is an essential enzyme activator, high Mn exposure disrupts the central nervous system, causes tumors and hypotonia, and impairs fetal development. The Mn content was above the permissible limit in all species studied and may pose a potential health risk if consumed frequently. Higher iron content was found in Voatsiperifery, Javan, and Sichuan peppers compared to all other species tested. Iron is responsible for the activity of some enzymes that produce energy. The presence of copper in the spice samples was found in a concentration range of 4.22 to 21.07 mg/kg. The limit value for copper in edible plants set by the FAO/WHO is 30.00 mg/kg [113]. Based on this recommendation, all spice samples analyzed in this study have copper content below the permissible limit. As an essential metal, Zn is responsible for the normal function of various types of enzymatic activity and the promotion of wound healing [111]. However, excessive intake or exposure to Zn can cause nausea, vomiting, abdominal pain, lethargy, and fatigue. In the present study, Javanese pepper had the highest zinc content and was above the permissible limit. The content of strontium was highest in Javanese pepper and that of rubidium in Bengal pepper. For all other species, the determined content was in the range of 9.90–95.41 mg/kg and 2.40–133.81 mg/kg, respectively. The lowest value for nickel in this study was found in Bengal pepper at 0.66 mg/kg and the highest at 3.05 mg/kg in Voatsiperifery pepper. Barium and chromium were not present in all samples, but, when they were detectable, they were highest in green and white pepper, respectively. The TXRF method also offers an interesting aspect that should be emphasized, namely the possibility of easily determining bromine, which is difficult to measure with other spectrometry techniques. In this study, the bromine levels were the lowest in white pepper (2.97 mg/kg) and highest in pink pepper (37.54 mg/kg). The results obtained provide a comparative basis for the evaluation of the nutritional value and quality control of spices and indicate the species-dependent bioaccumulation of essential trace elements.

## 3. Materials and Methods

All plant materials used (black pepper—Brazil, green pepper—India, white pepper—India, Melegueta pepper—Guinea, Voatsiperifery pepper—Madagascar, Javanese long pepper—Indonesia, pink pepper—Brazil, Bengali pepper—India, and Sichuan pepper—China) were purchased from a local spice store. Before hydrodistillation and solvent extraction, the plant material was ground in an electric mill (IKA-Werke GmbH & Co., Staufen im Breisgau, Germany).

### 3.1. Chemicals

All chemicals used are listed in Table 12. All chemicals were used without further purification.

### 3.2. Essential Oil Extraction

Essential oils were obtained from various plant materials via 2 h hydrodistillation using a Clevenger apparatus, employing 400 mL of distilled water for each sample. Due to the different content of essential oils, different masses of powdered paper were used. The specific masses used were as follows: 30 g of black pepper, green pepper, white pepper, Voatsiperifery pepper, pink pepper, and Sichuan pepper; 10 g of Javanese long pepper; 50 g of Bengali pepper; and 100 g of Melegueta pepper. Following extraction, the collected essential oil was dried with anhydrous Na_2_SO_4_ and stored in the dark at 4 °C, and its yield was calculated as a percentage (*v*/*w*):

(1) $$\mathrm{yield}=\frac{V}{m}\cdot 100$$

where *V* is the volume of the extracted essential oil in mL, and *m* is the mass of dry plant material used for extraction in g.

### 3.3. Solvent Extraction

First, 10.00 g of plant material was extracted with 70% EtOH in an ultrasonic bath for 20 min and filtered through Whatman paper No. 1 using a Büchner funnel. After filtration, the sample was re-extracted using the same procedure, the filtrates were combined, and the ethanol was removed under a rotary evaporator (Büchi Rotavapor, Flawil, Switzerland).

### 3.4. Characterization of Essential Oils and Ethanolic Extracts

The essential oils and ethanolic extracts were characterized using gas chromatography–mass spectrometry (GC-MS), Fourier-transform infrared spectroscopy (FTIR), high-performance liquid chromatography (HPLC), and nuclear magnetic resonance spectroscopy (NMR).

#### 3.4.1. Chemical Composition

The essential oils were analyzed by GC-MS using a 7890B gas chromatograph paired with a 5977A mass detector (Agilent, Santa Clara, CA, USA), featuring an HP-5ms capillary column (5% phenylmethylsiloxane, 30 m × 0.25 mm, film thickness 0.25 μm). The operating conditions included a carrier gas of helium at a flow rate of 1.2 mL/min. The temperature program consisted of an initial 2 min at 45 °C, followed by a rise to 250 °C at a rate of 4 °C/min and a final hold at 250 °C for 2 min. The injector temperature was set at 240 °C, with an injection volume of 1 μL and a split ratio of 1:100. Mass spectra were obtained at 70 eV, covering the range of 40–400 *m*/*z*. The components of the essential oil were identified by comparing their retention indices with literature values. The retention indices were established concerning a homologous series of n-alkanes (C9–C23) under identical operating conditions. The essential oil components were further identified by comparing their mass spectra with those stored in spectral data libraries or with literature mass spectra. The essential oil component quantities were assessed by normalizing the peak area.

#### 3.4.2. FTIR Spectrophotometry

The FTIR spectra of the essential oils and ethanolic extracts were obtained using a Bruker Vertex 70 spectrometer (Bruker Optik GmbH, Ettlingen, Germany) in FTIR-ATR mode, within the wavenumber range of 4000 to 400 cm^−1^ (MIR). Sixteen scans were obtained for each sample.

#### 3.4.3. NMR Spectrophotometry

All ^1^H NMR spectra were recorded using a Bruker Avance 600 NMR spectrometer (Karlsruhe, Germany) equipped with a 5 mm C/H dual probe and z-gradient accessory. The spectra were measured at 298 K with 128 scans, a relaxation delay of 10 s, a spectral width of 12,019 Hz, and an FID resolution of 0.37 Hz. The samples were dissolved in CDCl3, with TMS as an internal standard.

#### 3.4.4. Determination of Piperine by High-Performance Liquid Chromatography

The quantification of piperine in pepper fruits was performed according to the method described in the European Pharmacopoeia [114]. The plant material (0.250) was sonicated in 40 mL of ethanol (70%) for 20 min and filtered and diluted to 50.0 mL with the same solvent. Additionally, the sample was filtered through a membrane filter (nominal pore size: 0.45 µm). Quantification was performed using an Agilent 1260 Infinity II liquid chromatograph equipped with an autosampler, quaternary pump, column oven, and DAD detector (Agilent Technologies, Santa Clara, CA, USA). The mobile phase consisted of water (A) and acetonitrile (B) with the following gradient: 50% A from 0 to 5 min, 50–5% A from 5 to 20 min, 5–0% A from 20 to 22 min, with a flow rate of 1 mL/min. The analysis was performed using a Zorbax Eclipse XDB-C18 column (4.6 × 250 mm, particle size 5 µm, Agilent Technologies, Santa Clara, CA, USA). Piperine in the samples was identified by comparing the retention times and UV-Vis spectra with those of a standard piperine solution. Quantification was performed using calibration curves obtained by the linear regression analysis of six calibration points, y = 6092.1x + 18.552, R^2^ = 0.9999.

### 3.5. Antioxidant Activity

The antioxidant activity of the essential oils and ethanolic extracts was evaluated via the total phenolic content (TPC), total flavonoid content (TFC), and DPPH assay. A Shimadzu UV-1280 UV-Vis spectrophotometer (Shimadzu, Kyoto, Japan) was employed to determine total phenolics and flavonoids. The obtained results of the radical scavenging assay (DPPH) were compared with those of the commercial antioxidant BHT. TPC and TFC were expressed as gallic acid (mg GAE/mL) and rutin (mg RE/mL) equivalents, respectively.

The Folin–Ciocalteu method was employed to determine the total phenolic content of the essential oils and ethanolic extracts. The FC reagent was diluted with distilled water at a 1:9 volume ratio. All samples were diluted with methanol: 25 mL of the essential oil was introduced into a 10 mL volumetric flask and diluted to the mark with methanol; ethanolic extracts were diluted to create a methanolic solution with a concentration of 1 mg/mL. Next, 1 mL of the sample was combined with 5 mL of FC reagent, and, after a duration of 10 min, 4 mL of 7.5% aqueous Na_2_CO_3_ solution was introduced. The samples were incubated in the dark at room temperature for one hour, followed by absorbance measurement at 750 nm. A standard solution of gallic acid at a concentration of 2.5 mg/mL was prepared for the construction of the calibration curve. Solutions with concentrations ranging from 25 to 250 μg/mL were prepared through dilution with water. Reaction mixtures were prepared according to the procedure outlined for the essential oil and extract samples, and their absorbance was subsequently measured. A blank sample was created by using 1 mL of methanol instead of a sample.

The same diluted solutions of essential oils and ethanol extracts were used to determine the total flavonoid content, as in the FC method. First, 0.6 mL of a 5% (*w*/*v*) aqueous NaNO_2_ solution is added to 2 mL of sample. After 6 min, 0.6 mL of a 10% aqueous AlCl_3_ solution is added and mixed. After a further 6 min, 6.8 mL of aqueous NaOH solution (1M) is added. The reaction is carried out for 15 min in the dark at room temperature, after which the absorbance is measured at 510 nm. The same procedure was employed to prepare the calibration curve with a standard rutin solution (co = 0.58 mg/L). A blank sample was created by using 2 mL of methanol instead of a sample.

The radical scavenging capacity of the essential oils and ethanolic extracts was assessed utilizing a modified DPPH test. A 0.36 mM solution of the free radical DPPH was prepared by dissolving it in methanol. A 0.1 mL sample of the methanolic essential oil solution at several concentrations was combined with 0.1 mL of the DPPH solution on a 96-well plate and incubated in the dark at 25 °C for 30 min. A blank sample was created by combining 0.1 mL of methanol with the DPPH solution. The absorbance of each reaction mixture was measured at 517 nm (Chromate microplate reader, Awareness Technology, Inc., Palm City, USA).

### 3.6. Antimicrobial Activity

The antibacterial activity of the extracted compounds (black pepper, green pepper, white pepper, Melegueta pepper, Voatsiperifery pepper, Javanese pepper, pink pepper, Bengali pepper, and Sichuan pepper) was assessed against a panel of reference strains comprising Gram-positive *Enterococcus faecalis* (ATCC 29212) and *Staphylococcus aureus* (ATCC 25923) and Gram-negative *Escherichia coli* (ATCC 25925), *Pseudomonas aeruginosa* (ATCC 27853), and *Klebsiella pneumoniae* (ATCC 27736). Minimum inhibitory concentrations (MICs) were determined by the broth microdilution method in accordance with the CLSI M07 guidelines.

Assays were set up in sterile U-bottom 96-well microplates (Falcon 3077; Becton Dickinson, Franklin Lakes, NJ, USA) using cation-adjusted Mueller–Hinton broth (CAMHB containing Ca^2+^ and Mg^2+^; Becton Dickinson, Cockeysville, MD, USA). Twofold serial dilutions of each test sample were prepared across the plate to yield final concentrations of 512 to 0.25 mg/mL. The inoculum was prepared by creating a direct broth suspension of isolated colonies selected from an 18–24 h agar plate. The suspension was adjusted to turbidity equivalent to a 0.5 McFarland standard, yielding approximately 1–2 × 10^8^ CFU/mL. The optical density (OD) was determined at λ = 600 nm (OD_600_ ≈ 1.0) (Hach DR3900, Hach Lange GmbH, Düsseldorf, Germany). Sterile broth served as the control. Within 15 min of preparation, the adjusted suspension was diluted in broth so that, after inoculation, each tube contained approximately 5 × 10^5^ CFU/mL, as recommended by the CLS.

Following inoculation, the plates were incubated at 35 ± 2 °C for 16–20 h under ambient air. Growth was quantified spectrophotometrically at 600 nm using a BioSan microplate photometer (HiPo MPP-96) (Biosan, Riga, Latvia). The MIC was defined as the lowest concentration exhibiting no visible growth and a pronounced reduction in optical density relative to untreated control wells. All measurements were performed in duplicate to ensure the accuracy and reproducibility of the results. To assess the relative potency of the tested derivatives, we used ceftazidime (β-lactam) and ciprofloxacin (fluoroquinolone) as reference controls due to their clinical relevance, complementary mechanisms of action, and well-documented MIC ranges for the tested species, which allowed direct comparison with established susceptibility data. CAZ is highly active against Gram-negative bacteria but exhibits limited activity against Gram-positive organisms. In contrast, CIP, a fluoroquinolone, is also very effective against a broad range of Gram-negative species, while showing moderate activity against Gram-positive bacteria.

### 3.7. Trace Element Content

A stock solution of 1000 mg L1 (ROMIL PrimAg^®^ mono-component reference solutions, Cambridge, UK) was used to prepare the Ga internal standard solution. Ultrapure deionized water, used for the dilution of stock solutions and pepper samples, was obtained from a Milli-Q purifier system (Millipore Corp., Bedford, MA, USA). Nitric acid and hydrogen peroxide were used to digest pepper samples. A silicone solution in isopropanol (Serva GmbH & Co., Heidelberg, Germany) was used to coat all quartz glass disc reflectors in order to obtain a hydrophobic film so as to facilitate sample deposition.

Microwave acid digestion was used to prepare the pepper samples according to the method EPA 3052 [115]. The microwave oven used was a Speedwave XPERT (Milestone ETHOS EASY, Sorisole, Italy). About 100 mg of the sample was mixed with 7 mL of nitric acid and 1 mL of hydrogen peroxide in a polytetrafluoroethylene (PTFE) vessel. The vessels were sealed and heated following a two-step digestion program consisting of a first step for 5 min until 180 °C was reached and a second step of 10 min at 180 °C. After cooling, the digested sample solutions were transferred to a 15 mL flask and brought to volume with ultrapure deionized water. The experimental procedure for preparing pepper samples for TXRF measurements was as follows: 1.0 g pepper samples were spiked with 0.040 g of Ga 100 mg kg^−1^ (internal standard) to achieve a final concentration of 4 mg kg^−1^ of Ga. The mixture was homogenized by vortex agitation for 30 s, and an aliquot of a pepper sample of 10 μL was transferred onto a siliconized quartz glass sample carrier and dried using a hot plate set at a low temperature (T~40 °C). The measurement time was set at 600 s.

The TXRF analysis was performed using a commercial benchtop TXRF spectrometer, the S2 PICOFOX (BrukerNano, GmbH, Berlin, Germany), equipped with a low-power molybdenum X-ray tube (50 kV, 1 mA) and a silicon drift detector (SDD) with a resolution <150 eV at Mn-Kα. The evaluation of the TXRF spectra and the calculation of analyte net peak areas were performed using the software supplied with the equipment (Spectra Plus 7.8, Bruker AXS Microanalysis GmbH, Berlin, Germany).

## 4. Conclusions

This study analyzed the properties of essential oils and ethanolic extracts from nine pepper species to identify their chemical compositions and evaluate their biological activity. The TXRF technique was used for the first time to determine the microelement composition of pepper as part of this study. Javanese pepper exhibited the highest essential oil content, whereas Bengali and Sichuan peppers yielded the greatest amounts among the ethanolic extracts. The essential oil analysis by GC-MS, FTIR, and 1H NMR spectroscopy revealed the heterogenous compositions of the essential oils. The ethanolic extracts were assessed for their piperine content, with FTIR spectroscopy demonstrated to be a reliable, sustainable, and low-cost method for piperine monitoring in the tested pepper samples. Future research will be focused on developing an FTIR method for the quantitative analysis of the main bioactive marker, piperine, in the studied pepper species. Ethanolic extracts exhibited higher total phenolic and flavonoid content compared to essential oils, which was reflected in their enhanced antiradical and antimicrobial activity. Ethanolic extracts of Melegueta and Sichuan peppers demonstrated superior antioxidant activity, indicating their potential for application in the pharmaceutical and food industries. Ethanolic extracts of Javanese, Bengali, and Sichuan peppers demonstrated notable antimicrobial activity against *E. coli*, *P. aeruginosa*, and *S. aureus*, underscoring their potential as natural antimicrobial agents considering the growing concern over antimicrobial resistance. Future research should focus on optimizing the extraction parameters to obtain a composition that maximally enhances the antioxidant and antimicrobial activity of the tested pepper species.

## Figures and Tables

**Table 4:** Piperine content in ethanolic extracts—experimental data compared to published data.

Plant Material	Piperine Content, %		Refs.
	Exp	Lit	
Black pepper	3.10 ± 0.01	3.2–37.5	[58,74]
Green pepper	3.48 ± 0.03	5.09–28.32	[6,59]
White pepper	3.05 ± 0.02	0.92–51.92	[57,62]
Voatsiperifery pepper	0.86 ± 0.01	-	
Javanese pepper	-	0.03–1.12	[61,75]
Bengali pepper	3.38 ± 0.02	0.10–3.71	[56,75]

Literature values are given for the lowest and highest reported piperine content.

**Table 5:** Total phenolic (TPC) and flavonoid (TFC) content in EOs and EEs.

Pepper Species	TPC		TFC
	EOs, mg GAE/mL ^1^	EEs, mg GAE/g ^2^	EEs, mg RE/g ^2^
Black pepper	1.15 ± 0.08	93.87 ± 4.21	75.41 ± 7.31
Green pepper	0.96 ± 0.06	143.55 ± 13.53	264.98 ± 16.37
White pepper	0.86 ± 0.07	45.55 ± 2.05	221.53 ± 14.67
Melegueta pepper	0.29 ± 0.03	213.00 ± 14.51	346.78 ± 16.27
Voatsiperifery pepper	1.27 ± 0.05	36.16 ± 2.67	34.08 ± 5.24
Javanese pepper	1.59 ± 0.16	51.46 ± 3.99	60.28 ± 4.18
Pink pepper	1.29 ± 0.09	50.03 ± 3.89	19.81 ± 1.94
Bengali pepper	1.32 ± 0.10	31.46 ± 2.83	9.37 ± 0.21
Sichuan pepper	1.83 ± 0.03	162.63 ± 16.86	204.92 ± 18.76

GAE—gallic acid equivalents; RE—rutin equivalents; ^1^ mL of EO; ^2^ g of extract.

**Table 6:** Overview of the literature data on the topic of the TPC and TFC of pepper species essential oils (EOs) and ethanolic extracts (EEs).

Pepper Species	EO/EE	Extraction Method	Results (TPC, TFC, EC_50_, or AA)	Ref.
Black pepper	EO	HD	TPC = 237.556 mg GAE/g	[80]
		HD	TPC = 1.21 mg GAE/g	[24]
	EE	REX with 96% ethanol	TPC = 89.8 mg GAE/g TFC = 17.2 mg RE/g	[8]
		MAC, REX, UAE, SE with ethanol of various purities—results are given for 70% ethanol and UAE	TPC = 85.64 mg GAE/g TFC = 73.15 mg RE/g	[5]
		Shaking with 80% ethanol	TPC = 7.94 mg GAE/g TFC = 3.44 mg CE/g	[81]
		Shaking with 80% ethanol	TPC = 5.89–9.86 mg GAE/g TFC = 2.75–5.77 mg CE/g	[59]
		SE with ethanol	TPC = 62.3 mg CE/g	[60]
Green pepper	EO	HD	TPC = 0.4 mg GAE/g	[24]
	EE	Shaking with 80% ethanol	TPC = 14.15 mg GAE/g TFC = 9.23–10.83 mg CE/g	[59]
		MAC, UAE, MAE, UMAE with anhydrous ethanol—results are given for UAE	TPC = 3.75 mg GAE/g	[6]
White pepper	EO	HD	TPC = 0.94 mg GAE/g	[24]
Melegueta pepper	EO	HD	TPC = 5.45 mg GAE/L	[82]
	EE	MAC in ethanol (not specified)	TPC = 60.375 mg GAE/g	[83]
		MAC with ethanol of different purities; results are given for 70% ethanol	TPC = 90 mg GAE/g TFC = 65 mg GAE/g	[20]
Voatsiperifery pepper	EO	HD	TPC = 1.56 mg GAE/100 g	[29]
Javanese pepper	EE	REX with 96% ethanol	TPC = 75.6 mg GAE/g TFC = 4.5 mg RE/g	[8]
		MAC with 96% ethanol	TPC = 183.039 mg GAE/g TFC = 3.53 mg QE/g	[84]
		SE with ethanol	TPC = 123.1 mg CE/g	[60]
		MAC with 70 and 96% ethanol; results are given for 70% ethanol	TPC = 206.99 mg GAE/g TFC = 146.96 mg QE/g	[21]
Pink pepper	EE	SE and UAE with hexane, ethanol (>99.5%), and ethyl acetate, SFE; results are given for ethanol	TPC = 14.2 mg GAE/g	[19]
		SE, PLE with 99.9% ethanol	TPC = 23.26–54.85 mg GAE/g	[9]
		Shaking with 80% ethanol	TPC = 12.50–16.08 mg GAE/g TFC = 2.30–2.67 mg CE/g	[81]
		UAE with >95% ethanol	TPC = 22.29 mg GAE/g TFC = 3.93 mg QE/g	[55]
Bengal pepper	EE	MAC with 99.97% ethanol	TPC = 74.8 mg GAE/g TFC = 55.4 mg CE/g	[66]
		MAC with ethanol	TPC = 153.8 mg GAE/g	[85]

EO—essential oil; EE—ethanolic extract; HD—hydrodistillation; GAE—gallic acid equivalents; RE—rutin equivalents; QE—quercetin equivalents; CE—catechin or catechol equivalents; REX—reflux extraction; SE—Soxhlet extraction; UAE—ultrasound-assisted extraction; MAE—microwave-assisted extraction; UMAE—ultrasonic microwave-assisted extraction; MAC—maceration; SFE—supercritical fluid extraction; PLE—pressurized liquid extraction.

**Table 7:** EC_50_ values of EOs and EEs (DPPH assay).

Pepper Species	EC_50_, mg/mL	EC_50_, μg/mL
	EOs	EEs
Black pepper	68.75 ± 0.33	886.24 ± 22.85
Green pepper	47.29 ± 0.28	197.43 ± 11.48
White pepper	84.28 ± 5.65	1049.43 ± 64.78
Melegueta pepper	66.08 ± 6.62	96.33 ± 5.08
Voatsiperifery pepper	9.11 ± 2.86	1278.55 ± 139.53
Javanese pepper	25.57 ± 2.65	1127.85 ± 12.45
Pink pepper	8.45 ± 0.20	561.95 ± 82.83
Bengali pepper	8.78 ± 0.49	>12,300
Sichuan pepper	7.31 ± 0.08	99.37 ± 0.48

**Table 8:** Overview of the literature data on the topic of the antioxidant activity (DPPH assay) of pepper species’ essential oils (EOs) and ethanolic extracts (EEs).

Pepper Species	EO/EE	Extraction Method	Results (TPC, TFC, EC_50_, or AA)	Ref.
Black pepper	EO	Commercial oils	EC50 = 36.84 mg/mL	[38]
		HD	EC50 = 7.67 mg/mL	[24]
		UMAHD	EC50 = 6.348 mg/mL	[87]
		HD	EC50 = 1.15 mg/mL	[40]
		SD	EC50 = 4.18 mg/mL	[53]
	EE	REX with 96% ethanol	EC50 = 0.104 mg/mL	[8]
		MAC, REX, UAE, SE with ethanol of various purities—results are given for 70% ethanol and UAE	EC50 = 0.142 mg/mL	[5]
		SE with ethanol	EC50 = 14.15 μg/mL	[60]
Green pepper	EO	Commercial oils	EC50 = 38.77 mg/mL	[38]
		HD	EC50 = 40.86 mg/mL	[24]
	EE	MAC, UAE, MAE, UMAE with anhydrous ethanol—results are given for UAE	AA = 7.11.mg Trolox/mL	[6]
White pepper	EO	HD	EC50 = 45.02 mg/mL	[24]
		UMAHD	EC50 = 7.332 mg/mL	[87]
Melegueta pepper	EE	MAC in absolute ethanol	EC50 = 20.4 mg/mL	[98]
		40% ethanol; method not specified	EC50 = 150 μg/mL	[7]
		MAC in ethanol (not specified)	EC50 = 24.44 μg/mL	[83]
Javanese pepper	EO	HD	EC50 = 78.9 μg/mL	[44]
		HD	EC50 = 0.82 mg/mL	[40]
		HD	EC50 = 110 μg/mL	[46]
		HD	EC50 = 7.95 μL/mL	[47]
	EE	REX with 96% ethanol	EC50 = 0.378 mg/mL	[8]
		MAC with 96% ethanol	EC50 = 82.10 μg/mL	[84]
		SE with ethanol	EC50 = 10.54 μg/mL	[60]
Pink pepper	EO	HD	EC50 = 44.1 μg/mL	[49]
	EE	SE and UAE with hexane, ethanol (>99.5%), and ethyl acetate, SFE; results are given for ethanol	EC50 = 339 g/mL	[19]
		SE, PLE with 99.9% ethanol	AA = 108.33–317.14 μmol Trolox/g	[9]
		UAE with >95% ethanol	EC50 = 103.43 μmol Trolox/mL	[55]
Bengal pepper	EO	SD	EC50 = 0.6 mg/mL	[53]
Sichuan pepper	EO	MAHD	EC50 = 5 μL/mL	[99]

EO—essential oil; EE—ethanolic extract; HD—hydrodistillation; RE—rutin equivalents; REX—reflux extraction; SE—Soxhlet extraction; UAE—ultrasound-assisted extraction; MAE—microwave-assisted extraction; UMAE—ultrasonic microwave-assisted extraction; MAC—maceration; MAHD—microwave-assisted hydrodistillation; UMAHD—ultrasonic microwave-assisted hydrodistillation; SFE—supercritical fluid extraction; PLE—pressurized liquid extraction.

**Table 9:** Antimicrobial activity of selected pepper species’ essential oils against Gram-positive bacteria *S. aureus* and *E. faecalis* and Gram-negative bacteria including *E. coli*, *K. pneumoniae*, and *P. aeruginosa*.

Pepper Species	MIC, μg/mL				
	*Escherichia coli*	*Enterococcus* *faecalis*	*Klebsiella* *pneumoniae*	*Pseudomonas* *aeruginosa*	*Staphylococcus* *aureus*
Black pepper	128	256	128	128	64
Green pepper	128	>128	>64	128	>32
White pepper	128	>128	>64	64	>64
Melegueta pepper	128	>128	>64	>64	>64
Voatsiperifery pepper	128	>128	>64	>32	>64
Javanese pepper	128	>128	>64	>64	>64
Pink pepper	128	>128	>128	>64	>128
Bengali pepper	128	256	128	>64	>64
Sichuan pepper	128	256	128	>128	>128
CAZ	0.5	256	256	4	64
CIP	<0.125	0.5	256	<125	0.5

**Table 10:** Antimicrobial activity of selected pepper species’ ethanolic extracts against Gram-positive bacteria *S. aureus* and *E. faecalis* and Gram-negative bacteria including *E. coli*, *K. pneumoniae*, and *P. aeruginosa*.

Pepper Species	MIC, μg/mL				
	*Escherichia coli*	*Enterococcus* *faecalis*	*Klebsiella* *pneumoniae*	*Pseudomonas* *aeruginosa*	*Staphylococcus* *aureus*
Black pepper	128	256	128	64	128
Green pepper	128	256	128	128	128
White pepper	>64	256	128	128	>64
Melegueta pepper	>64	>128	256	>64	>64
Voatsiperifery pepper	>64	256	128	>64	>64
Javanese pepper	>8	>128	>32	>16	>8
Pink pepper	>16	>128	>64	>16	>32
Bengali pepper	>8	>128	>64	>64	>64
Sichuan pepper	>8	256	>32	>8	>32

**Table 11:** Trace element content of selected pepper species.

Concentration, mg/kg									
Element	White p.	Voats. p.	Javan. p.	Pink p.	Green p.	Black p.	Sichu. p.	Meleg. p.	Bengali p.
Cr	1.89	0.53	/	/	/	0.56	0.79	/	/
Mn	77.86	133.79	68.69	14.62	82.06	38.19	36.21	136.61	24.22
Fe	61.05	142.86	127.32	93.00	54.58	55.38	154.65	58.94	71.97
Ni	1.58	3.05	2.19	1.02	2.59	1.06	1.90	1.33	0.66
Cu	9.30	13.68	21.07	8.50	18.04	11.00	4.22	7.68	11.15
Zn	48.84	22.51	99.83	15.78	21.15	12.98	11.28	26.96	20.79
Br	2.97	4.81	5.79	37.54	6.12	28.65	4.06	4.62	12.80
Rb	2.40	65.00	72.13	29,13	31.17	21.45	4.37	16.46	133.81
Sr	14.68	31.94	95.41	37.54	52.08	24.19	27.62	9.90	56.25
Ba	4.40	4.81	33.61	/	55.71	/	/	/	27.84

/—not detected.

**Table 12:** List of chemicals.

Chemical	Manufacturer	CAS Number
DPPH ^1^, >97%	Fluka (Buchs, Switzerland)	1898-66-4
Gallic acid, 98%	Thermo Scientific (Waltham, MA, USA)	149-91-7
Folin–Ciocalteu reagent, 98%	VWR Chemicals (Radnor, PA, USA)	12111-13-6
BHT ^2^, 99%	Thermo Scientific (Waltham, MA, USA)	128-37-0
Aluminum chloride hexahydrate, >97%	BIOCHEM Chemopharma (Cosne-Cours-sur-Loire, France)	7784-13-6
Sodium carbonate, ≥99%	Lach-ner (Boston, MA, USA)	497-19-8
Sodium hydroxide, >98%	T.T.T. (London, UK)	1310-73-2
Sodium nitrite, >98%	Alkaloid Skopje (Skopje, North Macedonia)	7632-00-0
Sodium sulfate, 99.9%	Kemika d.d. (Zagreb, Croatia)	7757-82-6
Methanol, HPLC grade, 99.8%	J. T. Baker (Phillipsburg, NJ, USA)	67-56-1
n-Hexane, GC grade, ≥95%	Fisher Scientific (Hampton, NH, USA)	110-54-3
Rutin hydrate, >94%	TCI (Tokyo, Japan)	207671-50-9
Hydrogen peroxide, 30%, TraceSELECT	Sigma-Aldrich (St. Louis, MO, USA)	7722-84-1
Nitric acid, 69%, HIPERPUR	Panreac (Barcelona, Spain)	7697-37-2

^1^ 2,2-diphenyl-1-picrylhydrazyl. ^2^ butyl-hydroxytoluene.

## Data Availability

The original contributions presented in this study are included in the article/Supplementary Materials. Further inquiries can be directed to the corresponding author(s).

## Supplementary Materials

The following supporting information can be downloaded at: https://www.mdpi.com/article/10.3390/molecules30204140/s1. Table S1: Chemical composition of essential oils obtained by GC-MS.
