# Supplementary material for: Characterization of Essential Oils and Ethanolic Extracts from Nine Pepper Species: Antioxidant and Antimicrobial Activity and Spectroscopic Analysis
Source: Molecules. 2025 Oct 20;30(20):4140. doi: 10.3390/molecules30204140 (PMC12566369; doi:10.3390/molecules30204140)
Supplement: Supplementary file 1 [file molecules-30-04140-s001.zip › molecules-3923181-supplementary.pdf]

Table S1. Chemical composition of essential oils obtained by GC/MS

N	RI*exp	RIref	Compound	%								
				Black p.	Green p.	White p.	Bengal p.	Voatsip p.	Javan p.	Sichuan p.	Pink p.	Meleg. p.
1.	926	930	α-thujene	2.53	2.03	0.15	/	0.56	1.95	1.29	0.28	/
2.	933	939	α-pinene	6.12	5.54	7.22	0.28	6.39	1.52	4.69	15.46	/
3.	947	954	camphene	0.14	0.15	0.18	/	0.33	/	/	0.15	/
4.	973	975	sabinene	12.01	13.09	0.22	/	0.7	8.23	19.28	4.57	/
5.	977	979	β-pinene	8.83	7.04	11.23	0.23	5.03	0.32	1.16	5.75	/
6.	991	991	myrcene	1.67	1.71	2.52	/	1.02	0.21	7.17	4.93	/
7.	1005	1003	α-phellandrene	2.57	3.57	4.31	/	14.77	0.31	1.01	12.51	/
8.	1011	1010	δ-3-carene	9.79	11.78	21.89	/	6.43	/	/	15.98	/
9.	1017	1017	α-terpinene	0.82	0.25	/	/	0.13	/	2.08	0.2	/
10.	1024	1025	p-cymene	1.13	0.44	2.09	/	3.98	0.12	1.82	1.38	/
11.	1028	1030	β-phellandrene	/	/	/	/	/	1.75	20.83	/	/
12.	1029	1029	limonene	16.88	13.2	17.41	0.62	9.88	/	/	17.39	/
13.	1031	1031	1,8-cineole	/	/	/	/	0.51	0.66	6.75	/	/
14.	1037	1037	cis-β-ocimene	/	/	/	/	0.2	/	3.16	/	/
15.	1041	1044	2-heptanol-acetate	/	/	/	/	/	/	/	/	6.10
16.	1047	1050	trans-β-ocimene	/	/	/	/	0.14	/	1.27	/	0.17
17.	1057	1060	γ-terpinene	1.44	0.47	/	/	0.23	0.14	3.36	0.3	/
18.	1066	1070	cis sabinene hydrate	0.47	/	/	/	/	/	/	/	/
19.	1088	1089	terpinolene	0.82	0.74	0.79	/	0.35	/	1.46	0.87	/
20.	1099	1098	trans sabinene hydrate	0.39	/	/	/	/	/	/	/	/
21.	1101	1097	linalool	0.57	/	/	0.77	0.44	1.06	3.21	/	3.46
22.	1176	1177	terpinen-4-ol	2.58	0.72	/	/	0.43	0.25	7.71	0.4	/
23.	1186	1186	cryptone	/	/	/	/	/	/	0.68	/	/
24.	1190	1189	α-terpineol	0.31	/	/	/	0.27	/	1.89	/	/
25.	1253	1253	piperitone	/	/	/	/	0.52	/	3.66	/	/
26.	1256	1257	linalyl-acetate	/	/	/	/	/	/	0.44	/	/
27.	1287	1287	safrole	/	/	/	/	0.44	/	/	/	/
28.	1287	1292	6-tridecene	/	/	/	0.37	/	/	/	/	/
29.	1299	1300	tridecan	/	/	/	1.76	/	/	/	/	/
30.	1303	1299	carvacrol	/	/	/	/	0.34	/	/	/	/
31.	1336	1338	δ-elemene	0.39	0.62	2.18	/	0.19	0.33	/	0.36	/
32.	1348	1351	α-cubebene	/	/	/	/	0.26	2.46	/	/	/
33.	1369	1375	α-ylangene	/	/	/	/	0.39	/	/	/	/
34.	1374	1377	α-copaene	2.48	1.37	1.06	0.74	0.57	6.59	/	0.18	/
35.	1389	1388	β-cubebene	/	/	/	/	/	12.32	/	/	/
36.	1390	1391	β-elemene	0.43	0.94	/	1.99	0.7	/	/	0.34	/
37.	1405	1404	methyl-eugenol	/	/	/	/	3.01	/	/	/	/
38.	1407	1410	α-gurjunene	/	/	/	/	/	0.53	/	0.25	/
39.	1414	1414	cis-α-bergamotene	/	0.56	/	0.57	/	/	/	/	/
40.	1417	1419	β-caryophyllene	11.76	15.06	23.6	12.86	3.52	3.07	0.34	2.67	26.38

41.	1427	1432	β-copaene	/	/	/	/	0.4	/	/	/	/
42.	1434	1435	trans-α-bergamotene	/	0.65	/	0.97	/	/	/	/	/
43.	1451	1455	α-humulene	0.85	1.23	1.4	7.53	0.78	1.03	/	0.32	50.31
44.	1457	1457	cis-β-farnesene	/	0.82	/	1.58	/		/	/	/
45.	1458	1460	aromadendrene allo	/	/	/	/	/	3.73	/	/	/
46.	1475	1480	γ-muurolene	/	/	/	/	0.6	1.95	/	/	/
47.	1478	1485	germacrene D	/	0.5	/	10.18	2.3	6.77	0.34	4.46	/
48.	1482	1481	curcumene (ar)	/		/	1.33	/	/	/	/	/
49.	1483	1490	β-selinene	0.77	2.42	/	4.3	1.44	/	/	/	/
50.	1486	1488	cyclodecane, 1-ethyl-2-methyl	/	/	/	2.64	/	/	/	/	/
51.	1489	1494	trans-muurola-4(14),5-diene	/	/	/	/	0.19	0.27	/	/	/
52.	1492	1498	α-selinene	/	1,22	/	1,69	/	/	/	/	/
53.	1493	1500	bicyclogermacrene	/	/	/	/	/	5.47	/	0.47	/
54.	1494	1494	α-zingiberene	/	1.59	/	3.07	2.2	/	/	/	/
55.	1498	1492	trans-methyl-isoeugenol	/	/	/	/	0.86	/	/	/	/
56.	1498	1500	α-muurolene	0.34	/	/	/	/	0.88	/	0.26	/
57.	1500	1500	pentadecane	/	/	/	6.39	/	/	/	/	/
58.	1502	1507	α-bisabolene	/	/	/	1.69	/	/	/	/	/
59.	1503	1510	α-bulnesene	/	/	/	/	0.4	/	/	/	/
60.	1507	1506	β-bisabolene	2.49	2.75	/	6.17	2.06	/	/	/	/
61.	1513	1514	γ-cadinene	0.41	/	/	/	/	/	/	/	/
62.	1515	1522	epi-α-selinene	/	/	/	2.17	/	/	/	/	/
63.	1516	1515	cubebol	/	/	/	/	/	26.4	/	/	/
64.	1521	1519	myristicin	/	/	/	/	3.74	/	/	/	/
65.	1522	1512	δ-amorphene	/	0.92	/	/	/	/	/	/	/
66.	1522	1523	δ-cadinene	1.21	/	0.61	/	/	1.75	0.24	1.68	0.36
67.	1522	1525	β-sesquipellandrene	/	/	/	0.98	/	/	/	/	/
68.	1531	1531	trans-γ-bisabolene	/	1.21	/	0.86	/	/	/	/	/
69.	1542	1515	cis-γ-bisabolene	/	/	/	2.22	/	/	/	/	/
70.	1548	1550	elmol	4.52	3.13	/	/	0.7	/	/	2.23	/
71.	1554	1561	germacrene B	/	/	/	/	0.63	0.22	/	0.32	/
72.	1558	1557	elemicin	/	/	/	/	4.37	/	/	/	/
73.	1563	1563	*trans* nerolidol	0.66	0.69	/	/	0.22	1.21	/	/	1.31
74.	1575	1570	isoelemicin	/	/	/	/	1.26	/	/	/	/
75.	1580	1583	caryophillene oxide	0.37	0.28	0.87	2.62	0.52	/	/	/	2.83
76.	1596	1596	humulene epoxide I	/	/	/	/	/	/	/	/	0.69
77.	1606	1608	humulene epoxide II	/	/	/	/	/	/	/	/	5.18
78.	1615	1619	cubenol-1,10-di-epi	/	/	/	/	/	0.51	/	/	/

79.	1624	1621	dill apiol	/	/	/	/	3.22	/	/	/	/
80.	1629	1632	humulenol II	/	/	/	/	/	/	/	/	0.2
81.	1629	1632	γ-eudesmol	0.26	/	/	/	/	/	/	/	/
82.	1644	1646	α-muurolol	1.14	0.45	/	/	0.17	/	/	/	/
83.	1650	1654	α-eudesmol	0.50	/	/	/	/	/	/	/	/
84.	1669	1650	phlroacetophene dimethyl ether	/	/	/	/	/	/	2.64	/	/
85.	1681	1678	apiole					2.66	/	/	/	/
86.	1677	1673	tetradecanol-n	/	/	/	4.99	/	/	/	/	/
87.	1686	1682	1-heptadecene	/	/	/	8.47	/	/	/	/	/
88.	1699	1700	heptadecane	/	/	/	5.91	/	/	/	/	/
89.	1876	1876	hexadecanol-n	/	/	/	0.75	/	/	/	/	/

Kovats retention index determined relative to the retention time (tR) of a series of n-alkanes (C9–C23) on an HP-5ms capillary column. The identification of the compounds was performed based on the comparison of their relative retention time and mass spectra with those of the published data NIST, WILLY library data of the GC/MS system
